# Analysis of HIV Using a High Resolution Melting (HRM) Diversity Assay: Automation of HRM Data Analysis Enhances the Utility of the Assay for Analysis of HIV Incidence

**DOI:** 10.1371/journal.pone.0051359

**Published:** 2012-12-11

**Authors:** Matthew M. Cousins, David Swan, Craig A. Magaret, Donald R. Hoover, Susan H. Eshleman

**Affiliations:** 1 Department of Pathology, Johns Hopkins University School of Medicine, Baltimore, Maryland, United States of America; 2 Vaccine and Infectious Disease Division, Fred Hutchinson Cancer Research Center, Seattle, Washington, United States of America; 3 Department of Statistics and Biostatistics and Institute for Health, Health Care Policy and Aging Research, Rutgers University, Piscataway, New Jersey, United States of America; Queensland Institute of Medical Research, Australia

## Abstract

**Background:**

HIV diversity may be a useful biomarker for discriminating between recent and non-recent HIV infection. The high resolution melting (HRM) diversity assay was developed to quantify HIV diversity in viral populations without sequencing. In this assay, HIV diversity is expressed as a single numeric HRM score that represents the width of a melting peak. HRM scores are highly associated with diversity measures obtained with next generation sequencing. In this report, a software package, the HRM Diversity Assay Analysis Tool (DivMelt), was developed to automate calculation of HRM scores from melting curve data.

**Methods:**

DivMelt uses computational algorithms to calculate HRM scores by identifying the start (T1) and end (T2) melting temperatures for a DNA sample and subtracting them (T2–T1 = HRM score). DivMelt contains many user-supplied analysis parameters to allow analyses to be tailored to different contexts. DivMelt analysis options were optimized to discriminate between recent and non-recent HIV infection and to maximize HRM score reproducibility. HRM scores calculated using DivMelt were compared to HRM scores obtained using a manual method that is based on visual inspection of DNA melting curves.

**Results:**

HRM scores generated with DivMelt agreed with manually generated HRM scores obtained from the same DNA melting data. Optimal parameters for discriminating between recent and non-recent HIV infection were identified. DivMelt provided greater discrimination between recent and non-recent HIV infection than the manual method.

**Conclusion:**

DivMelt provides a rapid, accurate method of determining HRM scores from melting curve data, facilitating use of the HRM diversity assay for large-scale studies.

## Introduction

Accurate methods to estimate HIV incidence from cross-sectional surveys are needed for surveillance of the HIV/AIDS epidemic [1]. These methods could also be used to evaluate the effect of HIV prevention interventions in clinical trials [1]. Most methods for cross-sectional HIV incidence determination use serologic incidence assays to identify individuals with recent HIV infection, but these assays often overestimate incidence [2], [3], [4]. Alternative biomarkers for recent HIV infection are needed to improve the performance of HIV incidence testing algorithms. HIV diversity may be a useful biomarker for analysis of HIV incidence because levels of HIV diversity change during the course of HIV infection [5], [6].

We developed a rapid assay to quantify HIV diversity that does not require sequencing. This assay is based on high resolution melting (HRM) analysis [5], [7]. HRM scores are highly associated with diversity measures obtained using next generation sequencing [8]. Distinct patterns of HRM scores are associated with different stages of HIV disease, suggesting that the HRM diversity assay may be useful for analysis of HIV incidence [5]. While HRM assays typically measure small changes in the peak melting temperatures (Tm) of DNA amplicons to detect point mutations [9], this HRM diversity assay measures the width of the DNA melting peak to assess the level of diversity in a pool of DNA amplicons [7], [8], [10].

Plasma samples from individuals with recent and non-recent HIV infection were previously analyzed using the HRM diversity assay [5]. We used HRM data from that study to develop and optimize a software tool for automated calculation of HRM scores. Here, we describe the development and optimization of the HRM Diversity Assay Analysis Tool (DivMelt; available at: http://cran.r-project.org/web/packages/DivMelt/index.html), a software package that automates calculation of HRM scores from DNA melting curve data.

## Methods

### Source of HRM Data Used in the Analysis

Plasmids (n = 5) and plasma samples from individuals with acute (n = 20), recent (n = 102), and non-recent (n = 67) HIV infection were analyzed with the HRM diversity assay as part of a previous study [5]. We used HRM data from that study to develop and optimize DivMelt for automated calculation of HRM scores. A brief description of the HRM diversity assay is provided here for reference. HIV RNA is extracted from plasma or serum, reverse transcribed, and amplified using polymerase chain reaction (PCR). PCR products are purified and diluted for HRM analysis, which involves a second PCR reaction that includes a fluorescent dye. The resulting amplicons are analyzed using a LightScanner Instrument (Model HR 96, BioFire Diagnostics (formerly Idaho Technology), Salt Lake City, UT), which heats the sample and detects DNA melting based on release of a duplex-dependent fluorescent dye. The start (T1) and end (T2) melting temperatures can be determined from the raw melting data, and the difference between these two temperatures is the HRM score. In this study, samples were analyzed in duplicate, and the HRM scores from duplicate runs (ScoreA and ScoreB) were averaged. If the |ScoreA-ScoreB|/[(ScoreA+ScoreB)/2] was greater than 0.15, the data were excluded.

### Overview of DivMelt

**Figure 1:**
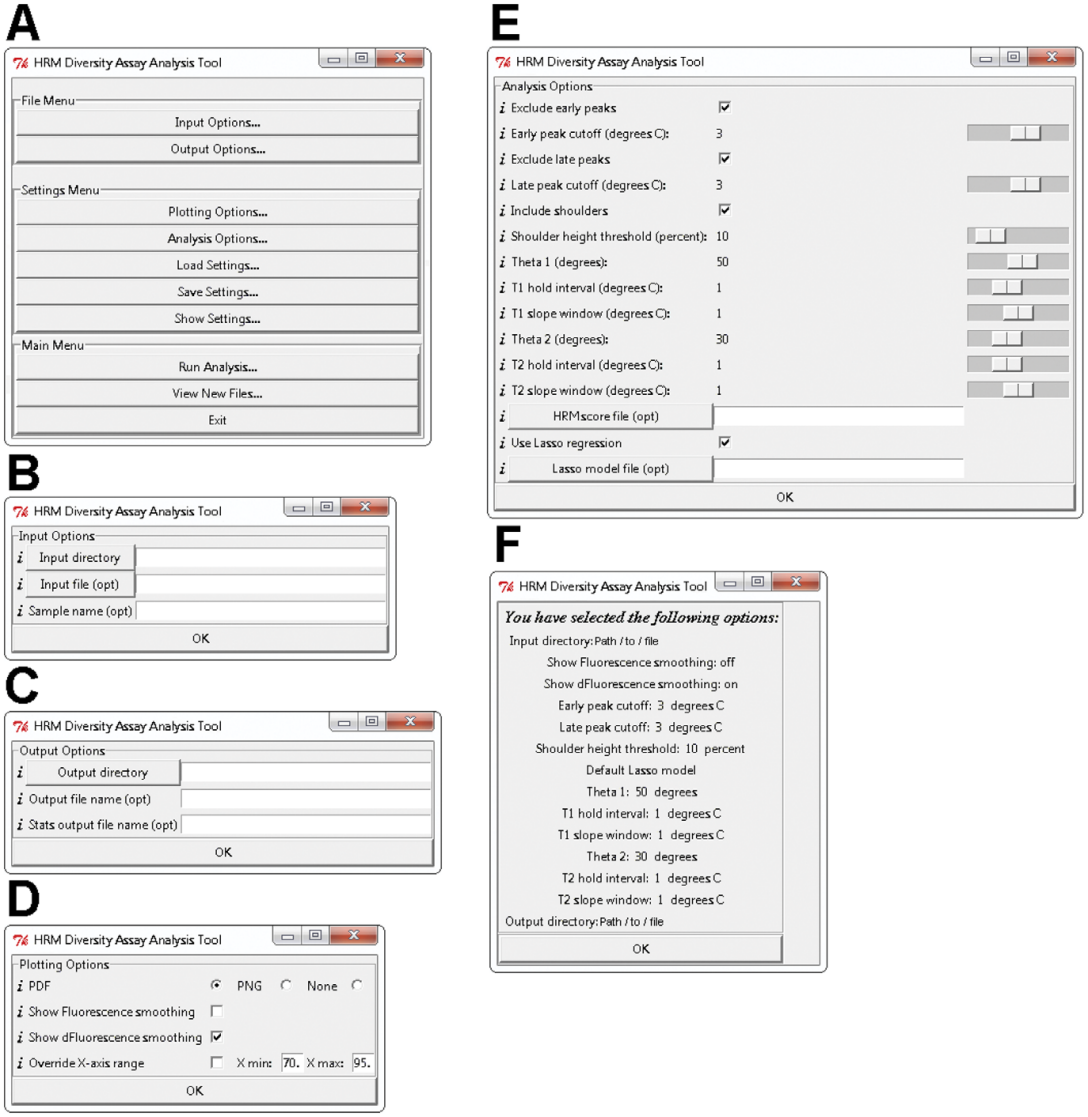
DivMelt Graphic User Interface (GUI). DivMelt displays various options when the package is opened. (A) The initial GUI window displays the “File Menu,” “Settings Menu,” and “Main Menu.” Sub-windows relating to file management may be opened from the “File Menu” window. These include (B) the “Input Options” window and (C) the “Output Options” window. From the “Settings Menu” window, additional options can be selected. These include (D) the “Plotting Options” window, (E) the “Analysis Options” window, and (F) the “Show Settings” window. The “Main Menu” is used to initiate analyses. Clicking “Run Analysis” opens the “Review Settings” window (similar to F). See the user manual for a description of the various tools accessible from the GUI. At the left of each row for items B–E, the “*i*” may be clicked to open a comment box that describes the function of the tool in question. These windows are shown as they display in Windows 7. Note that the windows will display slightly differently in other operating systems.

DivMelt is designed to analyze raw HRM data (Fluorescence vs. Temperature). DivMelt includes a graphical user interface (GUI) with multiple windows (Figure 1A–F). Using the GUI, the user may select from a menu of options for data input, data plotting, data analysis, and data output. The GUI is invoked when DivMelt is initiated in the R programming console.

**Figure 2:**
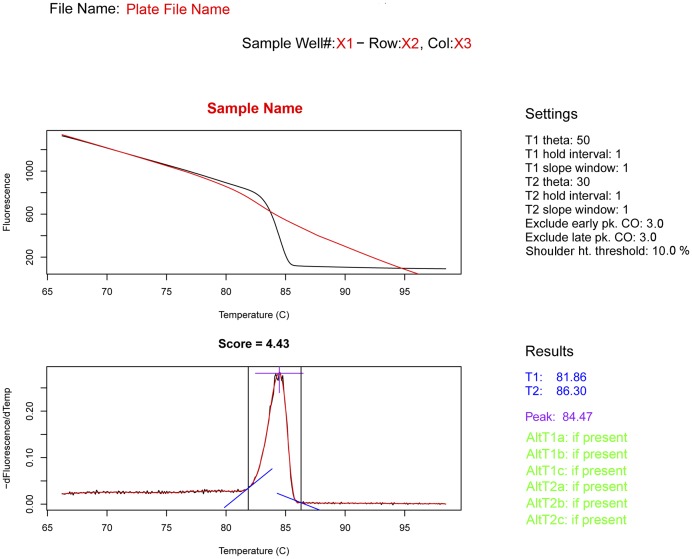
Fluorescence vs. Temperature and -dFluorescence/Temperature vs. Temperature plots. DivMelt generates PDF plots to allow visualization of the melting data. The plate file name is given at the top of each plot. Below the file name is the sample location information. This information includes the sample well number (X1; ranging 1–96), the row letter (X2; ranging A–H), and the column designation (X3; ranging 1–12). The sample name is presented in bold above the Fluorescence vs. Temperature plot (top panel). The Fluorescence vs. Temperature plot tracks the decline in fluorescence as the DNA melts in response to the temperature increase. To the right of this panel, the settings used in the analysis are noted. The lower panel consists of the melting peak or –dFluorescence/dT vs. Temperature plot. To the right of the lower panel are the analysis results (marked on the melting peak in equivalent colors). These include the T1 and T2 values (in blue) and the temperature that corresponds to the peak (in purple). Up to three alternative T1 and T2 values may be given if they are present in the analysis (in green). These alternatives are additional values that met the theta value criteria for T1 and T2 identification but were not selected by DivMelt. The HRM score (Score), the diversity output of the assay, is noted between the top and bottom panels of the figure. This value is calculated by subtracting T1 from T2 and corresponds to the peak width.

The input options allow the user to select from among three levels of raw HRM sample data for analysis: (1) a directory containing a series of data files generated from multiple 96-well plates, (2) a single data file generated from a single 96-well plate, or (3) a single sample from a single data file generated from a 96-well plate (Figure 1B). The output options allow the user to provide the file names and path destinations for the results files created by DivMelt (Figure 1C). The plotting options allow the user to generate PDF or PNG plots of the DNA melt data (Figure 1D). During analysis, the user may also choose to display the smoothed curves for the melting curve and melting peak, as described in a previous section. The X-axis range for these visualizations can be configured (Figure 1D). An example of the PDF plot format is provided in Figure 2.

The analysis options allow the user to tailor the analysis to the characteristics of a particular amplicon (Figure 1E). For detailed descriptions of each of the settings, see the Instruction Manual for the HRM Diversity Assay Analysis Tool (Document S1; http://code.google.com/p/divmelt/). A test dataset and dataset description have been made available (Archive S1; http://code.google.com/p/divmelt/); these materials can be used to test DivMelt and its features.

The GUI allows the user to configure the analysis settings for each session. The user may also load a settings file created during a previous analysis session, save a settings file for use in future analyses, or view the analysis settings currently selected (Figure 1A,F). Before a new analysis begins, the current settings are displayed for the user to review (window similar to Figure 1F).

By default, DivMelt generates a text file that contains all of the results generated during the analysis. The results are labeled by plate name, plate row, plate column, and sample name. These results include the calculated T1, T2, Tm, and HRM score values. Additionally, the DivMelt settings used for the analysis are included on each row. Alternative T1 and T2 values are assigned and listed in the analysis text file. Lastly, an acceptance/rejection decision is made based on the prediction of the sample's amplification success by the LASSO-based prediction model. The DivMelt features mentioned above will be described in greater detail in the following sections.

### Generation of Melting Peaks in DivMelt

**Figure 3:**
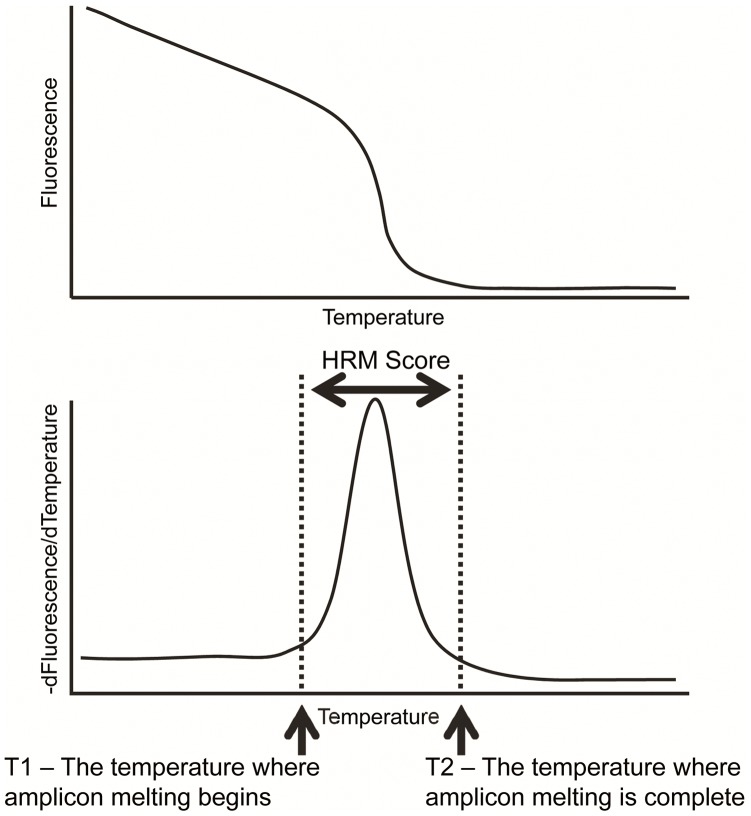
Generation of melting curves, melting peaks, and HRM scores. Melting curves (top panel) are generated by graphing Fluorescence against Temperature. Fluorescence declines as the DNA melts. DNA melting is visualized through the use of a saturating duplex-dependent DNA intercalating dye (LCGreen Plus). As the DNA melts, the dye is released; unbound dye does not fluoresce. Melting peaks (bottom panel) are generated by taking the negative derivative of Fluorescence with respect to Temperature and graphing these values against Temperature (−dF/dT vs T). T1 and T2 are identified on the melting peak, and these values are used to calculate the HRM score (T2–T1).

In DivMelt, the raw data (Fluorescence vs. Temperature) for each sample constitutes a raw melting curve (Figure 3). Data are plotted as the negative derivative of Fluorescence with respect to Temperature (−dF/dT) over Temperature ([−dF/dT]/T). These derivative values are smoothed with LOWESS (Locally Weighted Least Squares Scatterplot Smoothing) (Figure 3) [11], [12]. The model generated by LOWESS is defined as the melting peak, which is used in subsequent calculations. DivMelt was designed to improve upon a manual goniometer-based process of curve evaluation. The goniometer (a simple device used to measure angles) was previously used to identify the points on the DNA melting peak where the tangent to the melting peak first rose above or fell below a 30° angle with the baseline. The manual method requires data plots with an approximate aspect ratio of 2∶1 (X:Y). As a result, the data are scaled by DivMelt to a fixed 2∶1 aspect ratio for use in the construction of the derivative melting peaks ([-dF/dT]/T); this facilitates consistent trigonometric calculations. When HRM results are displayed on a computer monitor, the visual aspect ratio may vary from the 2∶1 established standard, but all internal calculations are performed with the data fixed at a 2∶1 aspect ratio.

### Rejecting PCR Failure Data

The HRM diversity assay is a PCR-based assay. As a result, there are occasional PCR failures (no or low amplification). A training set of sample data was used to assess the capacity of DivMelt to identify amplification failures (no amplification or sub-optimal amplification). The set contained data from samples that failed to amplify (n = 37) and samples that amplified successfully (n = 40). These data were used to generate the classification model that is used by DivMelt to identify samples that failed amplification. The values used by the classification model consist of summary statistics derived from the training set, including minimum, maximum, mean, and standard deviation of Fluorescence and -dFluorescence. The classification model was built from the training set with LASSO [13]; this model allows DivMelt to identify potential amplification failures and flag them for user review. To develop this model, PCR failures were first identified by viewing HRM melt curves; amplification failure was confirmed by analyzing the PCR products by agarose gel electrophoresis. HRM melt curves that resemble those obtained from samples that did not amplify properly are identified by DivMelt as PCR failures and are rejected. If desired, the user can substitute the default classification model with a custom model that is fitted to the user’s own data.

### Detection of Peak Temperature

While Tm is not used in the calculation of HRM scores, it is included in other applications of HRM technology. To detect the Tm, the melting peak is scanned with a sliding window of 2°C. DivMelt selects the point with the greatest absolute slope when looking to either side of the window’s mid-point. This value is analogous to the Tm value that is assigned by other HRM software applications. The peak value calculated by DivMelt may not be in complete agreement with Tm values from other software packages because this application does not control for linear and exponential background fluorescence [14], [15].

### Detection and Selection of T1 and T2 Values

**Figure 4:**
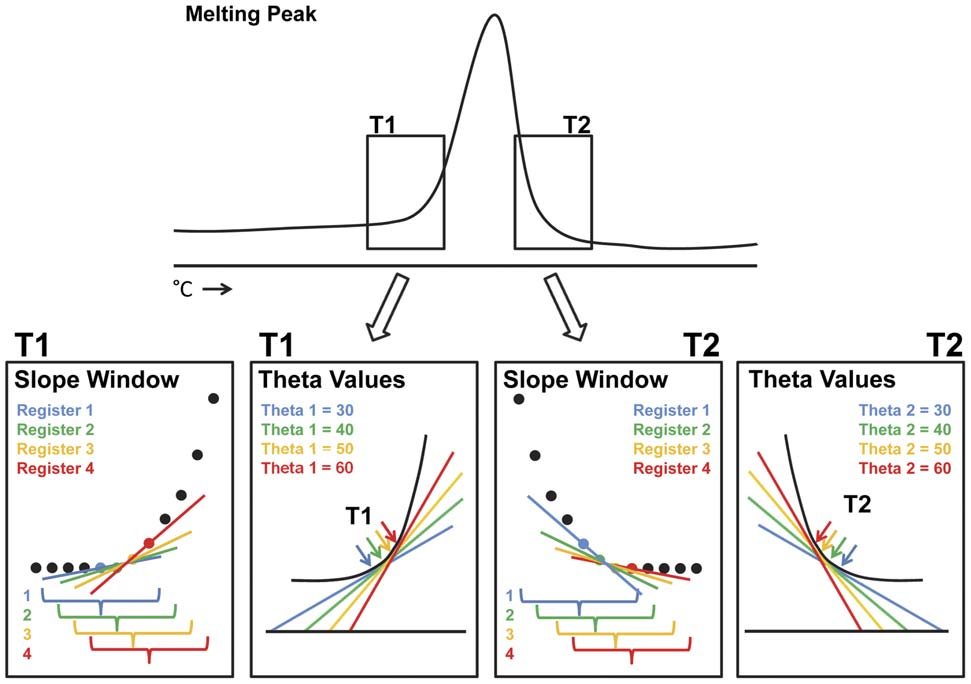
Methods for selection of T1 and T2 values. The slope of a line tangent to the melting peak is calculated for each point along the melting peak through the use of a sliding window (slope window). The user can define the width of this window. Each slope is compared with a user-defined Theta 1 value for selection of T1 and Theta 2 value for selection of T2. When the angle meets or exceeds Theta 1, T1 is identified. When the angle meets or exceeds Theta 2, T2 is identified.

T1 and T2 are the temperature values that correspond to the beginning and end of amplicon melting, respectively (Figure 3). The T1 value is selected by comparing the angle of a tangent to the smoothed curve line to a user-specified Theta 1 threshold (Figure 4). The T2 value is similarly selected through comparison of curve tangents with a Theta 2 threshold value. First, the melting peak is scanned with a sliding data window (slope window) of user-specified width (°C) (Figure 4), and the tangent angle to the local regression smoothing curve is calculated at every point along the smoothing line at each point beyond a threshold of 71°C. The angle at each point is calculated and compared with the user-specified Theta 1 value (Figure 4). When the tangent angle for a temperature value matches or exceeds Theta 1 on the low temperature side of the peak, that temperature is selected as the T1 value. Likewise, when the angle matches or falls below Theta 2 on the high temperature side of the peak, that temperature is selected as the T2 value (Figure 4).

DivMelt identifies all points that meet the angle (Theta 1 or Theta 2) threshold requirements for selection of T1 or T2. In the case where multiple potential T1 values are identified, DivMelt’s default behavior is to select the last (highest temperature) value as T1, and the remaining potential T1 values are identified as alternates for later consideration. Similarly, multiple potential T2 values may match T2 selection criteria. The default behavior of DivMelt is to select the first (lowest temperature) potential T2 value as T2, and the other potential T2 values are retained for further consideration.

DivMelt is highly user-configurable and includes options to minimize improper T1 and T2 selection. These parameters are described in detail in the Instruction Manual for the HRM Diversity Assay Analysis Tool (Document S1; http://code.google.com/p/divmelt/). Small, biologically irrelevant peaks that often appear as noise in the smoothed melting peak can cause DivMelt to assign an improper T1 or T2 value. The user can mitigate this improper selection with the “T1 hold interval” and “T2 hold interval” features by specifying the number of degrees Celsius over which Theta 1 or Theta 2 angle specifications must be met before T1 or T2 can be called.

A small subset of samples exhibit minor peaks that are discretely separated from the principal melting peak. These smaller peaks may occur at far lower temperatures or far higher temperatures (signifying non-amplicon material melting at much lower or higher temperatures). DivMelt allows the user to isolate the principal peak by excluding earlier peaks and later peaks based upon the number of degrees Celsius separating them from the principal peak. It is important that the user confirm that these peaks are superfluous by manual inspection before excluding them. Low-temperature minor peaks are believed to reflect the presence of primer dimers. The cause of high temperature secondary peaks is unknown.

**Figure 5:**
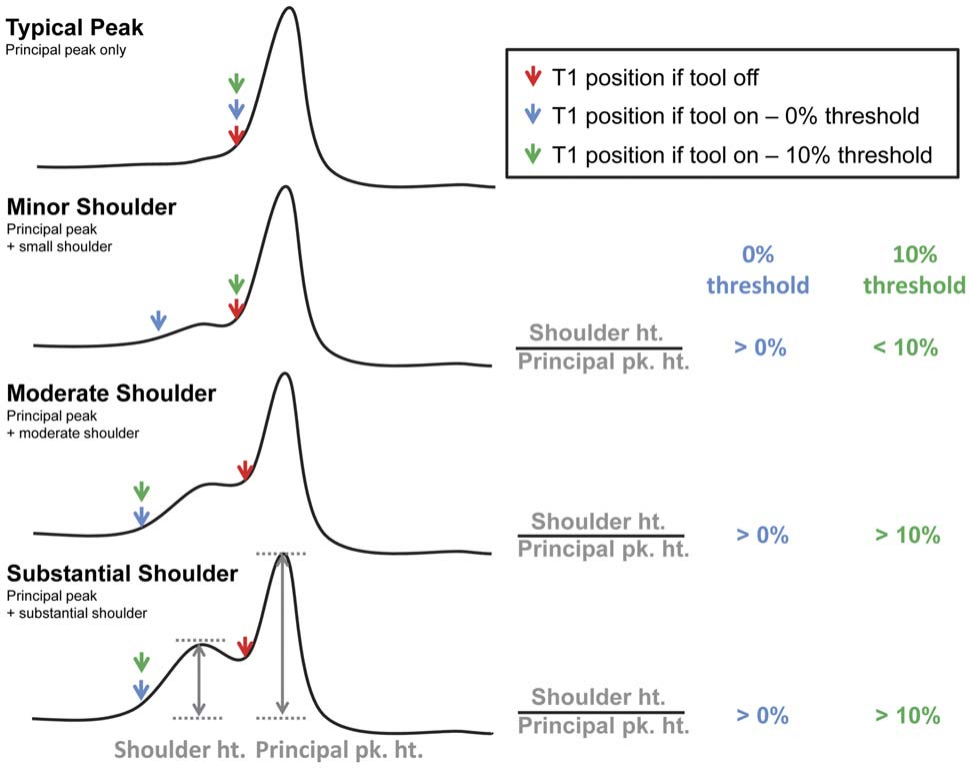
Description of the shoulder height threshold tool. The shoulder height threshold tool allows selective inclusion or exclusion of small peaks that are connected to the principal melting peak (shoulders). The image indicates the variation in T1 selection based on the specifications of the shoulder height threshold tool and the height of the shoulder.

Some melting peaks contain a minor peak abutting the low temperature side of the principal peak. This abutting peak is referred to as a “shoulder” and can give the melting peak a bimodal appearance (Figure 5). A user-controlled parameter (“include shoulders” tool) allows selective inclusion of these shoulders based on their height in relation to the height of the principal peak. When the height of the shoulder exceeds this user-specified value (given as a percentage of the principal peak height), the minor peak will be included, and the T1 value will be selected at the base of the shoulder (typically corresponding to a lower temperature). If the peak value of the shoulder does not exceed the user-specified percentage of the height of the principal peak, the T1 value will be called at the junction between the shoulder and the principal peak (Figure 5).

### Calculation of HRM Score

The HRM score is the difference between T1 and T2 (Figure 3). This is equivalent to the width of the primary melting peak described above. This measure represents the genetic diversity of the DNA analyzed.

### Assumptions Used in DivMelt Development

Some assumptions were made in the development of DivMelt in order to derive HRM score values from melting peak data. For example, it was assumed that the peak would have a steeper sloping high temperature side and a more variable slope for the low temperature side. These peak characteristics have been observed consistently in thousands of samples. Features were added to DivMelt to accommodate these assumed differences, helping identify the outer boundaries of the low temperature side of the peak (T1) versus the high temperature side of the peak (T2).

### Requirements for Running DivMelt

The R programming language is readily adaptable to many scientific and statistical situations [16]. DivMelt was designed to run as a package within the R computing environment. The package for calculating diversity measures from the HRM diversity assay is called “DivMelt: HRM Diversity Assay Analysis Tool” and can be downloaded from CRAN (http://cran.r-project.org/web/packages/DivMelt/index.html). Detailed documentation for package installation and operation instructions are included in the Instruction Manual for the HRM Diversity Assay Analysis Tool (Document S1; http://code.google.com/p/divmelt/). The package has been tested to run in R under Mac OS X, Windows 7, Windows XP, Windows Vista, and Linux, with only minor differences among operating systems. These differences are described in the instruction manual and documentation.

DivMelt is designed to accept data in the.ABT/.FLO format as generated by the LightScanner software package coupled to the LightScanner instrument. DivMelt is capable of accepting data generated from other software packages such as those that might be associated with RT-PCR systems that are occasionally used for HRM analysis; however, the output files would need to be reformatted.

### User Manipulation of DivMelt Results

DivMelt objectively calculates T1, T2, Tm, and HRM score values (Figure 2). DivMelt also records alternative T1 and T2 values in the event that the criteria for T1 and T2 selection are met at more than one temperature. The user can choose alternative T1 or T2 values if alternative values better represent the true temperature at which melting began or ended based on visual inspection of the melting curve. If the alternatives are selected, these temperature values and the resulting revised HRM score can be used in downstream analyses of the diversity results.

### Optimization of DivMelt

Many features were developed to allow DivMelt to function in different contexts. To optimize DivMelt for applications related to HIV incidence testing, DivMelt settings were optimized to distinguish between individuals with recent and non-recent HIV infection, as defined previously [5]. To enhance throughput, DivMelt was also optimized for reproducibility of HRM scores between duplicate analyses.

First, samples from adults with different stages of HIV infection were analyzed using the HRM diversity assay, and the results were analyzed with different DivMelt analysis parameters. Because T1 and T2 values should not have interacting impacts on HRM score, the selection of each of these values was optimized independently. Each temperature (T1 and T2) was optimized using a single analysis protocol for selection of the other temperature.

The parameters evaluated for T1 selection consisted of: Theta 1 values: 30, 40, 50, and 60; slope window widths of 0.5°C and 1°C; hold intervals of 0°C and 1°C; and shoulder tool: off, on set to 0%, and on set to 10% (Figures 3,4,5). Individual protocols were defined by the set of four parameters (Theta 1, slope window width, hold intervals, and shoulder tool). The parameters were used in all possible combinations, resulting in a set of 48 T1 analysis protocols with a fixed T2 selection (T2 protocol: Theta 2 = 30, slope window  = 1, and hold interval  = 0). For T2 selection, the following parameters were evaluated: Theta 2 values: 30, 40, 50, and 60; slope window widths of 0.5°C and 1°C; and hold intervals of 0°C and 1°C (Figure 4). The shoulder tool is not required for T2 optimization because T2 selection is not complicated by shoulders. As a result, only 16 T2 analysis protocols were evaluated using a fixed T1 protocol (T1 protocol: Theta 1 = 50, hold interval  = 0, slope window  = 1, and shoulder tool = on and set to 10%). HRM scores were analyzed to select optimal parameters to (1) distinguish between recent and non-recent infection and (2) maximize reproducibility of results, avoiding data rejection by quality control methodologies. Details of this analysis are provided in Document S2.

After independently identifying the best protocols for selection of T1 and T2, the optimal protocols for calculation of HRM score (being a combination of best T1 and best T2 protocols) were assessed for six different regions in the HIV genome (GAG1, GAG2, POL, ENV1, ENV2, and ENV3, defined in a previous report [5]). Results generated using the optimal (combined) protocols were (1) evaluated for ability to discriminate between recent and non-recent infection, (2) evaluated for reproducibility between duplicate runs, (3) correlated with results from the manual method, (4) scored for the ability to properly recognize T1, T2, and Tm values, and (5) verified for proper detection of amplification failure samples.

### Statistical Methods

HRM score data were found to be most normally distributed with the most equality of variance between recent and non-recent samples when data were subjected to a double log transformation. To identify the “best” protocol for selection of T1 or T2 for an individual genomic region, normalized distance (ND) measures were used to compare the degree to which the various analysis protocols distinguished between recent and non-recent HIV infection. The ND statistic was calculated by the following formula using double log transformed data: ND = (U_NR_–U_R_)/SQRT([σ^2^_R_+σ^2^_NR_]/2) where R = recent, NR = non-recent, U = mean (as estimated by the sample), and σ^2^ =  variance (as estimated by the sample). Values were then ranked according to ND.

The highest ranked T1 or T2 selection protocols by ND were then compared with regard to the number of test samples excluded by the reproducibility quality control protocol. All samples were analyzed in duplicate, and the HRM scores from duplicate runs (ScoreA and ScoreB) were averaged. If the |ScoreA-ScoreB|/[(ScoreA+ScoreB)/2] was greater than 0.15, the data were excluded. We first defined the minimum number of samples excluded by any protocol for each region. The “best” protocol was defined as the highest ranked protocol as determined by having the maximum ND that excluded fewer than “minimum” +3 samples for T1 and equal to the “minimum” for T2. Further details of this analysis are provided in Document S2.

The best T1 selection protocols and T2 selection protocols were combined to identify the optimal analysis protocols. The optimal analysis protocols for each specific region were evaluated for ND, data exclusion, correlation with manually obtained data (Pearson’s correlation coefficient), and proper identification of T1, T2, and Tm values by visual scoring. A protocol capable of analyzing data from all 6 regions simultaneously was also identified based on a combination of ND ranks from each of the 6 regions with an emphasis on minimizing data exclusion due to failure to meet the quality control threshold. Analyses were performed using SAS version 9.2 (Carey, NC).

## Results

DivMelt was developed for rapid and objective processing of HRM data, and it was designed around a user-friendly GUI that is invoked through the R program (Figure 1). DivMelt generates easily interpretable graphics representing melting curves and melting peaks (Figure 2), along with text files for use in downstream analyses. During DivMelt development, specific features of DNA melting peaks were identified, and various user-specified parameters were added to DivMelt to allow customization of analyses based upon specific features associated with each amplicon being analyzed. The development of these options provided flexibility and allowed for the optimization of DivMelt for discrimination between recent and non-recent HIV infection and for reproducibility of HRM scores generated from duplicate runs.

DivMelt contains a LASSO regression model that is used to identify PCR failures so that these data can be excluded from downstream analyses. This feature successfully identified 54 instances of amplification failure regardless of the analysis settings specified in DivMelt. It also mistakenly identified 14 (0.6%) of 2,328 successful PCR amplifications as failures.

To optimize DivMelt to distinguish between recent and non-recent HIV infection, HRM scores were calculated for six different regions of the HIV genome from 169 individuals with different stages of HIV infection (102 recent and 67 non-recent). Analyses were performed using 48 different protocols for selection of T1 (while the T2 selection protocol was held constant) and 16 different protocols for selection of T2 (while the T1 selection protocol was held constant) (Figure 3). HRM scores from each selection protocol were then examined to identify the best T1 and T2 selection protocols for distinguishing between recent and non-recent HIV infection according to ND analysis; higher ND values indicated greater discrimination between recent and non-recent infection (Document S2).

Among the top T1 selection protocols for each region, the highest ranked protocol that avoided excessive sample data exclusion from a set of 189 samples (20 acute, 102 recent, and 67 non-recent) was identified as the best T1 selection protocol. Data from acute infection samples was incorporated into this analysis to provide a larger amount of data, increasing the strength of conclusions. The same process was conducted for T2 to identify the best T2 selection protocol. The process for identification of these protocols is described in detail in Document S2.

After the best protocols for selection of T1 and T2 were identified, these methods were used in combination, yielding optimal analysis protocols for calculation of HRM scores (see Table 1 for details). The optimal analysis protocols were then compared for their ability to discriminate between recent and non-recent infection (Table 2). The optimal analysis protocols for each genomic region had higher ND values than the corresponding values obtained using manually-generated HRM scores. This indicates that DivMelt is superior to the manual method for discriminating between recent and non-recent HIV infection. The ENV2 region provided the least discrimination between recent and non-recent infection in a previous study where HRM scores were generated manually [5]. However, the ability to discriminate between these two groups using ENV2 data was increased when DivMelt was used for analysis (use of DivMelt was associated with an increase in the ND from 0.41 to 1.02). The optimal analysis protocols also excluded relatively few samples due to our quality control threshold (∼1% or less in all cases) (Table 3), and the majority of the optimal DivMelt analysis protocols yielded results that were strongly correlated with manual HRM score data, indicated by r values >0.85 for GAG1, GAG2, POL, ENV1, and ENV3 (Table 4). However, the optimal ENV2 protocol had a lower r value of 0.62, indicating a weaker correlation. In addition to identifying region-specific optimal analysis protocols, a single robust analysis protocol that worked well for all six regions was identified. This protocol performed similarly to the manual method for discrimination between recent and non-recent infection and did not exclude any data due to the data quality control threshold.

**Table 1:** Optimal DivMelt analysis protocols for discrimination between recent and non-recent HIV infection.

Region Used for Optimization a	T1				T2		
	Theta 1	HI	SW	SH	Theta 2	HI	SW
GAG1	30	1	1	10%	60	0	0.5
GAG2	40	1	1	10%	40	0	1
POL	60	1	0.5	10%	50	0	0.5
ENV1	60	1	0.5	off	40	0	0.5
ENV2	60	1	1	10%	40	0	0.5
ENV3	30	0	1	0%	60	0	1
All regions	50	1	1	10%	40	0	1

Definitions and abbreviations: T1– temperature when melting began; T2– temperature when melting was complete; Theta 1– theta angle (°) for selection of T1; Theta 2– theta angle (°) for selection of T2; HI – hold interval (°C); SW – slope window (°C); SH - Shoulder ht threshold (%) tool (“off” indicates that the shoulder height threshold tool was not in use).

a HIV genome regions (amplicons examined) have been described previously [5]. Briefly, the amplicons and the proteins coded by the corresponding HIV genomic regions are as follows: GAG1, p7; GAG2, p6 and transframe; POL, protease and reverse transcriptase; ENV1, HR1 region of gp41; ENV2, immunodominant region of gp41; and ENV3, HR2 region of gp41.

**Table 2:** Identification of the optimal DivMelt analysis protocols for discriminating between recent and non-recent HIV infection as measured by normalized distance^a^.

Region Used for Optimization b	Region Analyzed c					
	GAG1	GAG2	POL	ENV1	ENV2	ENV3
GAG1	**1.46**	1.34	1.55	0.98	0.28	1.74
GAG2	1.36	**1.54**	1.56	1.32	0.24	1.66
POL	1.06	1.10	**1.82**	1.47	1.11	0.96
ENV1	0.76	0.97	1.06	**1.62**	0.74	0.58
ENV2	0.98	1.20	1.73	1.49	**1.02**	1.03
ENV3	1.35	1.18	1.47	1.09	0.31	**1.84**
Manual HRM Score	1.21	1.44	1.47	1.47	0.42	1.72
All regions	1.32	1.27	1.78	1.43	0.41	1.63

a Reported values are normalized distance between the mean of recent infection samples and the mean of non-recent infection samples. The region used to optimize each protocol is shown in the column on the left; the regions analyzed are shown in the headers for the six other columns.

b Detailed region-specific analysis protocol descriptions are shown in Table 1.

c HIV genome regions are described in the footnote of Table 1.

**Table 3:** Percentage of samples excluded by the internal quality control for each of the optimal DivMelt analysis protocols^a^.

Region Used for Optimization b	Region Analyzed c					
	GAG1	GAG2	POL	ENV1	ENV2	ENV3
GAG1	**0.53**	5.82	0.00	0.53	0.00	0.00
GAG2	0.53	**0.00**	0.00	0.00	2.12	0.00
POL	1.06	2.65	**0.53**	0.00	1.06	0.00
ENV1	0.00	2.12	1.06	**0.53**	1.59	0.53
ENV2	0.00	1.06	0.53	0.53	**1.06**	0.00
ENV3	0.00	6.88	0.00	1.59	1.06	**1.06**
All regions	0.00	0.00	0.00	0.00	0.00	0.00

a All values are reported as percentages. The region used to optimize each protocol is shown in the column on the left; the regions analyzed are shown in the headers for the six other columns.

b Detailed region-specific analysis protocol descriptions are shown in Table 1.

c HIV genome regions are described in the footnote of Table 1.

**Table 4:** Correlation between HRM scores calculated with the optimal DivMelt analysis protocols and HRM scores calculated using the manual method a.

Region Used for Optimization b	Region Analyzed c					
	GAG1	GAG2	POL	ENV1	ENV2	ENV3
GAG1	**0.94**	0.77	0.96	0.85	0.37	0.88
GAG2	0.98	**0.95**	0.97	0.93	0.57	0.89
POL	0.83	0.80	**0.95**	0.94	0.67	0.63
ENV1	0.52	0.74	0.70	**0.88**	0.48	0.38
ENV2	0.76	0.83	0.90	0.90	**0.62**	0.63
ENV3	0.90	0.68	0.95	0.84	0.34	**0.87**
All regions	0.96	0.90	0.95	0.96	0.84	0.94

a Pearson’s correlation coefficient with manual HRM score. The region used to optimize each protocol is shown in the column on the left; the regions analyzed are shown in the headers for the six other columns.

b Detailed region-specific analysis protocol descriptions are shown in Table 1.

c HIV genome regions are described in the footnote of Table 1.

To validate DivMelt for analysis of DNA melting curves, the accuracy of T1, T2, and Tm calculations was scored for the optimal protocol for each region. The Tm was correctly identified in all 189 samples for all six regions analyzed and in all duplicate runs (n = 2,268 total melting curves) for all of the optimal protocols. T1 was correctly identified >99% of the time for the optimal protocols, and T2 was correctly identified >99% of the time in the optimal protocols for all regions except for GAG2 (∼98.7%) (see Document S3 for details).

## Discussion

DivMelt facilitates rapid and objective analysis of HRM data to generate HIV diversity measures (HRM scores). DivMelt is designed to run in the R computing environment. This application can also be easily adapted to quantitatively assess other aspects of HRM-derived DNA melting peaks. To our knowledge, DivMelt is the first publically available software application for quantification of complex features of HRM-derived DNA melting curves.

In the past, we analyzed HRM diversity assay data using a goniometer to identify the start and end of DNA melting. This manual method was time-consuming, subjective, and dependent on the format of the computer monitor. In contrast, DivMelt is objective and is not sensitive to monitor display format (aspect ratio). DivMelt also analyzes data approximately 50–100 times faster than is possible with the manual method. The quality of the text files generated allows for rapid downstream processing of the data to generate HRM scores. Using optimized DivMelt settings, HRM scores obtained for duplicate runs were highly reproducible. This minimized exclusion of sample data and increased the efficiency of the HRM diversity assay. Visual inspection of the DNA melting curves confirmed that DivMelt accurately identified T1, T2, and Tm values. These findings demonstrate that DivMelt is a robust tool for analysis of DNA melting curves.

We are attempting to determine whether HIV diversity can be used as a biomarker for cross-sectional HIV incidence determination [5]. Most likely, the HRM diversity assay would be used as the last step in a multi-assay incidence algorithm (i.e., to identify individuals who are misclassified as recently infected using low cost, high-throughput serologic incidence assays) [5]. For this reason, we optimized DivMelt for maximum discrimination between recent and non-recent HIV infection. The DivMelt software program also reduces the time and effort needed for HRM diversity analysis, which facilitates analysis of multiple regions of the HIV genome in large sample sets. Because viral suppression is associated with misclassification by serologic incidence assays, some multi-assay incidence algorithms include HIV viral load and classify samples with viral suppression as “non-recent”. A lack of amplification in the PCR used to prepare templates for the HRM diversity assay may serve as surrogate for low viral load, particularly if multiple regions fail to amplify; therefore, inclusion of HRM diversity analysis in an incidence-testing algorithm may eliminate the need for HIV viral load testing.

Using optimized software settings, DivMelt provided better discrimination between recent and non-recent HIV infection than did the manual method for calculating HRM scores. The ability to discriminate between recent and non-recent infection using DivMelt varied among the six different HIV genomic regions examined, consistent with results obtained using a manual method; this indicates that diversity in different regions of the HIV genome may be more or less informative as a biomarker for HIV incidence determination.

Optimized DivMelt settings provide an approximation of the manual method of melting curve analysis. However, the data from these two methods should not be directly compared. Four previous studies used the manual method for analysis of HRM data [5], [7], [10], [17], and two recent studies used DivMelt [8], [18]. DivMelt provides better discrimination between recent and non-recent HIV infection and generates data in a more objective, rapid, and robust fashion.

DivMelt is a publicly available software application developed to enhance the throughput and validity of HRM diversity assay results. The availability of DivMelt facilitates the use of the HRM diversity assay for HIV incidence testing. DivMelt also increases the potential utility of the HRM diversity assay for biologic studies. This approach for automated melting curve analysis can also be applied to other systems that require detailed quantitative analysis of DNA melting peaks and their features.

## Supporting Information

Archive S1 Test dataset for analysis with the HRM Diversity Assay Analysis Tool (DivMelt). Artificial diverse DNA populations were created by mixing mutagenized and wildtype HIV-derived plasmids. The diverse DNA populations were amplified and analyzed with the HRM diversity assay. These data files were exported from the LightScanner Instrument and Analysis Software (BioFire Diagnostics, Salt Lake City, UT) package and serve as input data for DivMelt.
(ZIP)

Document S1 Instruction Manual for the HRM Diversity Assay Analysis Tool (DivMelt). Detailed DivMelt instructions and software feature descriptions are provided in this document.
(PDF)

Document S2 Identification of the best HRM Diversity Assay Analysis Tool (DivMelt) protocols for selection of T1 and T2. A large number of different software analysis protocols were evaluated for selection of T1 and T2 (48 protocols for determination of T1 and 16 protocols for determination of T2). The best T1 and T2 selection protocols were those that maximized discrimination between recent and non-recent infection while also minimizing data exclusion due to quality control measures.
(PDF)

Document S3 Accuracy of T1 and T2 values identified using the HRM Diversity Assay Analysis Tool (DivMelt). T1 and T2 temperatures identified with DivMelt were visually scored for accuracy.
(PDF)
