# Supplementary material for: Analysis of HIV Using a High Resolution Melting (HRM) Diversity Assay: Automation of HRM Data Analysis Enhances the Utility of the Assay for Analysis of HIV Incidence
Source: PLoS One. 2012 Dec 11;7(12):e51359. doi: 10.1371/journal.pone.0051359 (PMC3519891; doi:10.1371/journal.pone.0051359)
Supplement: Archive S1 — Test dataset for analysis with the HRM Diversity Assay Analysis Tool (DivMelt). Artificial diverse DNA populations were created by mixing mutagenized and wildtype HIV-derived plasmids. The diverse DNA populations were amplified and analyzed with the HRM diversity assay. These data files were exported from the LightScanner Instrument and Analysis Software (BioFire Diagnostics, Salt Lake City, UT) package and serve as input data for DivMelt. (ZIP) [file pone.0051359.s001.zip › Archive_S1/DivMelt_test_dataset_description.pdf]

**Test Dataset for Analysis with the HRM Diversity Assay Analysis Tool (DivMelt)**

Summary

The three files contained in the directory along with this document constitute a test dataset for the HRM Diversity Assay Analysis Tool (DivMelt). These high resolution melting (HRM) data were generated from artificial diverse DNA populations that were created by mixing mutagenized and wildtype HIV-derived plasmids. The plasmids used to generate this dataset were the subject of an article that is currently in press.

Cousins MM, Donnell D, Eshleman SH (2012) Impact of mutation type and amplicon characteristics on genetic diversity measures generated using a high resolution melting diversity assay. Journal of Molecular Diagnostics. In Press.

Preparation of files

The HRM diversity assay was run as previously described (Cousins et al., 2011, PLoS ONE, 6(11):e27211), and a 96-well plate was imaged with the LightScanner instrument (BioFire Diagnostics, Salt Lake City, UT). Following analysis with the LightScanner, melt data were exported from the LightScanner Instrument and Analysis Software package as .ABT, .FLO, and .TEM files. The .ABT file contains sample name information used by DivMelt. The .FLO file contains melting data (fluorescence and temperature data) used by DivMelt. The .TEM file contains temperature data and is not used by DivMelt but is included here for completeness.

DivMelt features illustrated by the test dataset

The analysis of this dataset will illustrate several key features of DivMelt. The data were selected to provide the opportunity to observe package functionality in multiple situations. A detailed description of the test dataset is provided in the summary table given on pages 2-3 of this document.

1. The dataset contains data from 4 assay runs (wells on the 96-well plate) that were identified as marginal amplification failures by DivMelt. These assay runs are identified by the LASSO regression model that is contained within DivMelt.

2. Data from no template controls (NTC) were also included. The software tool automatically screens out wells labeled with “NTC”, and these wells are not analyzed.

3. Each sample assayed in this test dataset was assayed in duplicate. This allows for comparisons to be made between duplicate assay runs as a measure of reproducibility.

4. The assay was performed on samples that were known to have certain diversity features. Clear increases in melting peak width are noted when mutations are introduced into amplicon pools (when shorter amplicons are examined). More significant mutations cause greater increases in peak width (9 bp insertion > 3 bp insertion > single base change > 1 bp deletion).

5. The examination of longer amplicons allows observation of more complex melting peak characteristics and melting domains. DivMelt functions in these contexts, but increases in peak width due to the presence of mutations are less easily observed when longer amplicons are assayed.

6. Analysis of this data with different user-specified analysis protocols demonstrates the functionality of the various DivMelt analysis options.

**Test Data Summary Table.** Samples analyzed to generate the test dataset.

| Well # | Designation | Mixture Status | Mutation Type | Amplicon Length |
|---|---|---|---|---|
| 1,9 | WT_100bp_POL | Pure WT | N/A | 100 bp |
| 2,10 | WT+AtoG(50:50)_100bp_POL | 50% WT + 50% mutant | SBC (WT+AtoG) | 100 bp |
| 3,11 | WT+AtoC(50:50)_100bp_POL | 50% WT + 50% mutant | SBC (WT+AtoC) | 100 bp |
| 4,12 | WT+AtoT(50:50)_100bp_POL | 50% WT + 50% mutant | SBC (WT+AtoT) | 100 bp |
| 5,13 | WT+del1bp(50:50)_100bp_POL | 50% WT + 50% mutant | 1 bp deletion | 100 bp |
| 6,14 | WT+ins3bp(50:50)_100bp_POL | 50% WT + 50% mutant | 3 bp insertion | 100 bp |
| 7,15 | WT+ins9bp(50:50)_100bp_POL | 50% WT + 50% mutant | 9 bp insertion | 100 bp |
| 8,16 | NTC | | | |
| 17,25 | WT_200bp_POL | Pure WT | N/A | 200 bp |
| 18,26 | WT+AtoG(50:50)_200bp_POL | 50% WT + 50% mutant | SBC (WT+AtoG) | 200 bp |
| 19,27 | WT+AtoC(50:50)_200bp_POL | 50% WT + 50% mutant | SBC (WT+AtoC) | 200 bp |
| 20,28 | WT+AtoT(50:50)_200bp_POL | 50% WT + 50% mutant | SBC (WT+AtoT) | 200 bp |
| 21,29 | WT+del1bp(50:50)_200bp_POL | 50% WT + 50% mutant | 1 bp deletion | 200 bp |
| 22,30 | WT+ins3bp(50:50)_200bp_POL | 50% WT + 50% mutant | 3 bp insertion | 200 bp |
| 23,31 | WT+ins9bp(50:50)_200bp_POL | 50% WT + 50% mutant | 9 bp insertion | 200 bp |
| 24,32 | NTC | | | |
| 33,41 | WT_300bp_POL | Pure WT | N/A | 300 bp |
| 34,42 | WT+AtoG(50:50)_300bp_POL | 50% WT + 50% mutant | SBC (WT+AtoG) | 300 bp |
| 35,43 | WT+AtoC(50:50)_300bp_POL | 50% WT + 50% mutant | SBC (WT+AtoC) | 300 bp |
| 36,44 | WT+AtoT(50:50)_300bp_POL | 50% WT + 50% mutant | SBC (WT+AtoT) | 300 bp |
| 37,45 | WT+del1bp(50:50)_300bp_POL | 50% WT + 50% mutant | 1 bp deletion | 300 bp |
| 38,46 | WT+ins3bp(50:50)_300bp_POL | 50% WT + 50% mutant | 3 bp insertion | 300 bp |
| 39,47 | WT+ins9bp(50:50)_300bp_POL | 50% WT + 50% mutant | 9 bp insertion | 300 bp |
| 40,48 | NTC | | | |
| 49,57 | WT_400bp_POL | Pure WT | N/A | 400 bp |
| 50,58 | WT+AtoG(50:50)_400bp_POL | 50% WT + 50% mutant | SBC (WT+AtoG) | 400 bp |
| 51,59 | WT+AtoC(50:50)_400bp_POL | 50% WT + 50% mutant | SBC (WT+AtoC) | 400 bp |
| 52,60 | WT+AtoT(50:50)_400bp_POL | 50% WT + 50% mutant | SBC (WT+AtoT) | 400 bp |
| 53,61 | WT+del1bp(50:50)_400bp_POL | 50% WT + 50% mutant | 1 bp deletion | 400 bp |
| 54,62 | WT+ins3bp(50:50)_400bp_POL | 50% WT + 50% mutant | 3 bp insertion | 400 bp |
| 55,63 | WT+ins9bp(50:50)_400bp_POL | 50% WT + 50% mutant | 9 bp insertion | 400 bp |
| 56,64 | NTC | | | |
| 65,73 | WT_600bp_POL | Pure WT | N/A | 600 bp |
| 66,74 | WT+AtoG(50:50)_600bp_POL | 50% WT + 50% mutant | SBC (WT+AtoG) | 600 bp |
| 67,75 | WT+AtoC(50:50)_600bp_POL | 50% WT + 50% mutant | SBC (WT+AtoC) | 600 bp |
| 68,76 | WT+AtoT(50:50)_600bp_POL | 50% WT + 50% mutant | SBC (WT+AtoT) | 600 bp |
| 69,77 | WT+del1bp(50:50)_600bp_POL | 50% WT + 50% mutant | 1 bp deletion | 600 bp |
| 70,78 | WT+ins3bp(50:50)_600bp_POL | 50% WT + 50% mutant | 3 bp insertion | 600 bp |
| 71,79 | WT+ins9bp(50:50)_600bp_POL | 50% WT + 50% mutant | 9 bp insertion | 600 bp |
| 72,80 | NTC | | | |

**Test Data Summary Table Continued.** Samples analyzed to generate test dataset.

| Well # | Designation | Mixture Status | Mutation Type | Amplicon Length |
|---|---|---|---|---|
| 81,89 | WT_800bp_POL | Pure WT | N/A | 800 bp |
| 82,90 | WT+AtoG(50:50)_800bp_POL | 50% WT + 50% mutant | SBC (WT+AtoG) | 800 bp |
| 83,91 | WT+AtoC(50:50)_800bp_POL | 50% WT + 50% mutant | SBC (WT+AtoC) | 800 bp |
| 84,92 | WT+AtoT(50:50)_800bp_POL | 50% WT + 50% mutant | SBC (WT+AtoT) | 800 bp |
| 85,93 | WT+del1bp(50:50)_800bp_POL | 50% WT + 50% mutant | 1 bp deletion | 800 bp |
| 86,94 | WT+ins3bp(50:50)_800bp_POL | 50% WT + 50% mutant | 3 bp insertion | 800 bp |
| 87,95 | WT+ins9bp(50:50)_800bp_POL | 50% WT + 50% mutant | 9 bp insertion | 800 bp |
| 88,96 | NTC | | | |

Abbreviations: Well # – well number on the 96-well plate; WT – wild type; SBC – single base change; bp – base pair; NTC – no template control; AtoG – a base that was “A” in the WT plasmid was changed to “G”; AtoC – a base that was “A” in the WT plasmid was changed to “C”; AtoT – a base that was “A” in the WT plasmid was changed to “T”
