# Supplementary material for: Analysis of HIV Using a High Resolution Melting (HRM) Diversity Assay: Automation of HRM Data Analysis Enhances the Utility of the Assay for Analysis of HIV Incidence
Source: PLoS One. 2012 Dec 11;7(12):e51359. doi: 10.1371/journal.pone.0051359 (PMC3519891; doi:10.1371/journal.pone.0051359)
Supplement: Document S1 — Instruction Manual for the HRM Diversity Assay Analysis Tool (DivMelt). Detailed DivMelt instructions and software feature descriptions are provided in this document. (PDF) [file pone.0051359.s002.pdf]

Instruction Manual for the HRM Diversity Assay Analysis Tool (DivMelt)

Matthew M. Cousins[1], David Swan[2], Craig A. Magaret[2], Susan H. Eshleman[1]

[1]Department of Pathology, Johns Hopkins School of Medicine, Baltimore, MD, 21205
[2]Statistical Center for HIV/AIDS Research & Prevention (SCHARP), Fred Hutchinson Cancer Research Center, Seattle, WA 98109

Contents

Section	*Page(s)*
Section I: Introduction	1-2
Section II: Installation	3-6
Section III: Preparation of data for analysis	7
Section IV: Analysis of data	8-10
Section V: DivMelt options, features, and outputs	11-17
Section VI: References	18
Section VII: Figures and captions	19-28

SECTION I: Introduction

Background

The HRM diversity assay allows rapid quantification of DNA diversity without sequencing [1]. The assay employs analysis of DNA melting curves like those generated in other high resolution melting applications (Figure 1). The melting patterns of these fluorescently labeled nucleic acid populations, specifically the widths of the DNA melting peaks (Figure 2), indicate the levels of diversity in the DNA populations. The peak width has been termed the HRM score. This diversity measure has been previously validated through comparisons between melting data results and sequence diversity derived through a traditional sequence strategy as well as diversity measures derived from next generation sequencing data [2]. Additionally, higher diversity was observed in individuals who had been infected with HIV for longer periods of time. HIV is known to diversify over time after infection [1].

Assay Overview

The HRM diversity assay is based upon the principle that DNA heteroduplexes generated from diverse DNA pools will melt over broader temperature ranges than heteroduplexes generated from less diverse DNA pools. Initially, highly variable nucleic acid pools are amplified via PCR (polymerase chain reaction) in the presence of a duplex-dependent fluorescent dye (LCGreen Plus; Figure 3). Following amplification, a rapid cooling step leads to the formation of fluorescently labeled heteroduplexes. The fluorescently labeled DNA heteroduplexes are subsequently melted, and a highly sensitive camera is used to detect the loss of fluorescence as a function of DNA melting (Figure 3). A melting curve (Fluorescence vs. Temperature; Figure 1) is generated by graphing Fluorescence against Temperature. By taking the negative derivative of Fluorescence with respect to Temperature (-dF/dT) and graphing this against Temperature, a melting peak is generated. The width of the melting peak corresponds to the HRM score, the diversity output of the HRM diversity assay (Figure 3). In the past, HRM score values have been generated by first selecting the temperature where melting begins (T1) and the temperature where melting is complete (T2) by a manual method. A goniometer was used to

select the point where melting begins and is complete while the melting peak was displayed within the LightScanner Instrument and Analysis Software package [BioFire Diagnostics (formerly Idaho Technology), Salt Lake City, UT] (Figure 4). However, this approach has proven to be time consuming, subjective, dependent upon the monitor format, and dependent upon X-axis settings within the program. A standardized and objective method for data analysis is required for analysis of melting curves. The HRM Diversity Assay Analysis Tool (DivMelt) was created to automate the calculation of diversity measures from lab-generated datasets.

Introduction to DivMelt

DivMelt rapidly and objectively calculates diversity measures from HRM data. In general, the calculation is performed in four steps. First, the Fluorescence by Temperature data is modeled (melting curve; Figure 1). Second, the negative derivative of Fluorescence with respect to Temperature is calculated and modeled against Temperature (melting peak; Figure 2; black line), and a smoothing line is generated (Figure 2; red line). Third, the temperature at which melting starts and finishes is identified. This is performed by scanning the melting peak for the point where the -dF/dT vs. T smoothing line first rises above a specified angle (Theta 1) and falls below a specified angle (Theta 2) to identify T1 and T2 respectively. Fourth, by subtracting T1 from T2, the peak width is calculated (Figure 2). The peak width is the HRM score. This single numeric score serves as a measure of the level of genetic diversity in the sample that was evaluated.

Disclaimer

The HRM Diversity Assay is for research purposes only and is not suitable as a diagnostic test. DivMelt is provided without any warranty and may be distributed freely as long as its use is appropriately cited.

When using DivMelt, please cite as follows:

Cousins MM, Swan D, Magaret CA, Hoover DR, Eshleman SH (2012) Analysis of HIV Using a High Resolution Melting (HRM) Diversity Assay: Automation of HRM Data Analysis Enhances the Utility of the Assay for Analysis of HIV Incidence. PLoS ONE. In Press.

SECTION II: Installation

This section describes the installation process for DivMelt. This program has been designed to run within the R computing environment. As a result, this installation includes details as to the R installation process. Additionally, this protocol describes the acquisition of additional packages that are required to run DivMelt and the installation of DivMelt itself. These processes are detailed for Windows and MacOS X.

To install DivMelt in Windows:

Note: This installation protocol has been tested for Windows 7 and Windows Vista.

1. Visit the R project website (http://www.r-project.org/).

2. Under the “Getting Started:” heading, click “download R.”

3. Select the CRAN mirror nearest your location.

4. Click "Download R for Windows."

5. Click "base."

6. Click "Download R X.XX.X for Windows.”

   Note: DivMelt was developed to run in R version 2.14.0.

7. Install R by following the on screen prompts.

8. Open the R GUI (Graphic User Interface). This interface can be opened from a shortcut on the desktop (if one was generated during installation) or from a location within the “Start” menu.

9. Under the “Packages” heading, click “Install Packages…” This will open a “CRAN mirror” selection window. You should select the CRAN mirror nearest your location.

10. A “Packages” window will open after a CRAN mirror is selected. In order to run DivMelt, two R packages must first be installed.

11. Select the “glmnet” package, and click “OK” to install this package.

12. You will be asked to create a personal library to install packages. Click "OK." The package will be installed.

13. Following the same protocol, select the “tcltk2” package, and click “OK” to install this package. The package will be installed.

14. Download the DivMelt package. The package may be downloaded from the CRAN website (http://cran.r-project.org/).

15. Place the package file, “DivMelt_X.X.X.tar.gz,” in the directory containing the R executable file. The default Windows 7 location is “C:\Program Files\R\R- X.XX.X \bin\i386” for R

version X.XX.X. This will vary with the R version installed and the Windows version that is installed on your machine.

16. Open the Windows Command Prompt. This can be done by opening the “Run” window, entering “cmd”, and pressing “Enter.”

17. Enter the following text: cd “C:\Program Files\R\R- X.XX.X \bin\i386” (the quotes should be included) or the location of the R executable file (R.exe) on your machine and press "Enter." The following text will be printed: “C:\Program Files\R\R-X.XX.X\bin\i386>” (this may vary somewhat depending on the location of the R executable file and the version of R installed).

18. Enter “R.exe CMD INSTALL DivMelt_X.X.X.tar.gz”, and press the “Enter” key to install the package (do not include the quotes in this statement).

    Text from Windows Command Prompt follows (may vary depending on the Windows version and the location of the R.exe file):

    Note: Text that is user-provided will appear in bold.

    Microsoft Windows [Version 6.1.7601]
    Copyright <c> 2009 Microsoft Corporation. All rights reserved.

    C:\Users\username>**cd “C:\Program Files\R\R-X.XX.X\bin\i386**

    C:\Program Files\R\R- X.XX.X \bin\i386>**R.exe CMD INSTALL DivMelt_X.X.X.tar.gz**
    * installing to library ‘C:/Program Files/R/R-X.XX.X/library’
    * installing *source* package ‘DivMelt’ …
    ** R
    ** data
    ** preparing package for lazy loading
    Warning: replacing previous import ‘head’ when loading ‘utils’
    Warning: replacing previous import ‘tail’ when loading ‘utils’
    ** help
    *** installing help indices
    ** building package indices …
    ** testing if installed package can be loaded
    Warning messages:
    1: replacing previous import ‘head’ when loading ‘utils’
    2: replacing previous import ‘tail’ when loading ‘utils’

    * DONE (DivMelt)

    Note: The text “username” in the above text string will need to be changed to the username appropriate for the user and the machine that is being used to conduct the analysis.

To install DivMelt in MacOS X:

Note: This installation protocol has been tested for MacOS X 10.6.

1. Visit the R project website (http://www.r-project.org/).

2. Under the “Getting Started:” heading, click “download R.”

3. Select the CRAN mirror nearest your location.

4. Click "Download R for MacOS X."

5. Click "R-X.XX.X.pkg" (latest version). This should be the latest version of R.

   Note: DivMelt was developed to run in R version 2.14.0.

6. Install R by following the on screen prompts.

7. Open R from the “Applications” menu.

8. Under the “Packages and Data” heading, click “Package Installer.” This will open a “CRAN mirror” selection window. You should select the CRAN mirror nearest your location. A “Package Installer” window will open after a CRAN mirror is selected. In order to run DivMelt, two R packages must first be installed.

9. Select the “glmnet” package (hold command key).

10. Select the “tcltk2” package (while still holding the command key).

11. Click “Install Selected” to install the packages.

12. Download “tcltk utilities” from the R Project website (http://cran.r-project.org/bin/macosk/tools/).

13. Install “tcltk utilities” package.

14. Open R and go to "Packages and Data" → "Package Manager" to load packages.

15. Click on “glimnet” to load. Successful loading will be noted in the R graphic user interface.

16. Click on “tcltk” to load. Successful loading will be noted in the R graphic user interface.

17. Download DivMelt, and save the package to the desktop. The package may be downloaded from the CRAN website (http://cran.r-project.org/).

18. Open terminal.

19. Type the following command: “R CMD INSTALL /Users/username/Desktop/DivMelt_X.X.X.tar.gz” (no quotes), and press “Enter.” There must be a space between the word “INSTALL” and “/Users/…” in the above text.

    Text from Macintosh Terminal follows (may vary depending on the MAC OS version and the location of the R.exe file):

    Note: Text that is user-provided will appear bolded.

    computername:~ username $ **R CMD INSTALL /Users/username/Desktop/DivMelt_X.X.X.tar.gz**
    * installing to library ‘/Library/Frameworks/R.framework/Versions/2.14/Resources/library’
    * installing *source* package ‘DivMelt’...
    ** R
    ** data
    ** preparing package for lazy loading
    Warning: replacing previous import ‘head’ when loading ‘utils’
    Warning: replacing previous import ‘tail’ when loading ‘utils’
    ** help
    *** installing help indices
    ** building package indices...
    ** testing if installed package can be loaded
    Warning messages:
    1: replacing previous import ‘head’ when loading ‘utils’
    2: replacing previous import ‘tail’ when loading ‘utils’

    * DONE (DivMelt)

    Note: The text “username” and “computername” in the above text string will need to be changed to the username and computername appropriate for the user and the machine that is being used to conduct the analysis.

SECTION III: Preparation of Data for Analysis

The program can analyze melt data collected from any instrument designed to measure a decline in fluorescence over an increase in temperature; however, this data must be in the proper format. Many real-time PCR systems are capable of generating suitable data, but the data files generated may not be properly formatted for analysis using this program. This software package was designed to accept data outputs from the LightScanner Instrument and Analysis Software (BioFire Diagnostics, Salt Lake City, UT).

To prepare data for analysis:

1. Open the “LightScanner Instrument and Analysis Software.”

2. Open the plate file to be analyzed.

3. Click: “File” → ”Export” → ”Export current subset as FLO file” (this will open a “save as” window; See Figure 5).

4. Save the export file set. It is suggested that the export file be saved into a directory on the desktop.

   Note: This will result in the generation of three text files in the chosen directory. Each file has a different extension.
   .FLO – Contains temperature and fluorescence values for each sample well.
   .ABT – Contains sample names and sample well designations.
   .TEM – Contains temperature values from the analysis.

5. Delete the “.TEM” file as this file type is not used by DivMelt.

SECTION IV: Analysis of Data

To open DivMelt:

1. Open the R GUI from the “Start” menu (Windows), a desktop shortcut (Mac or Windows), or the “Applications” menu (Mac).

2. In the R console:
   Type “library(DivMelt)” and press “Enter.”
   Type “DivMelt_gui ()” and press “Enter.”

   Resulting text in “R Console” is given below (User-provided text is noted in bold; may vary slightly based on R version):

   R version 2.14.0 (2011-10-31)
   Copyright (C) 2011 The R Foundation for Statistical Computing
   ISBN 3-900051-07-0
   Platform: i386-pc-mingw32/i386 (32-bit)

   R is free software and comes with ABSOLUTELY NO WARRANTY.
   You are welcome to redistribute it under certain conditions.
   Type 'license()' or 'licence()' for distribution details.
     Natural language support but running in an English locale
   R is a collaborative project with many contributors.
   Type 'contributors()' for more information and
   'citation()' on how to cite R or R packages in publications.

   Type 'demo()' for some demos, 'help()' for on-line help, or
   'help.start()' for an HTML browser interface to help.
   Type 'q()' to quit R.

   > **library(DivMelt)**
   Loading required package: tcltk
   Loading Tcl/Tk interface ... done
   Loading required package: glmnet
   Loading required package: Matrix
   Loading required package: lattice

   Attaching package: ‘Matrix’
   The following object(s) are masked from ‘package:base’:
       det
   Loaded glmnet 1.7.1

   Warning messages:
   1: replacing previous import ‘head’ when loading ‘utils’
   2: replacing previous import ‘tail’ when loading ‘utils’
   > **DivMelt_gui()**
   <Tcl>
   >

Note: the HRM GUI will open after this process is complete (Figure 6).

Note: After loading the HRM library in the R GUI by typing "library(DivMelt)" and pressing "Enter," the user may view documentation describing the main graphic user interface of DivMelt by typing "?DivMelt_gui" into the R GUI and pressing "Enter."

To analyze data with DivMelt:

Note: See the section "DivMelt Options, Features, and Outputs" for detailed descriptions of the functions of each GUI element.

Note: After loading the DivMelt library in R GUI by typing "library(DivMelt)" and pressing "Enter," the user may view documentation describing the main routine of DivMelt by typing "?processDivMelt" in the R GUI and pressing "Enter."

1. Select an input directory by clicking the "Input Options" button then the "Input directory" button and selecting a folder from the navigation window. This folder will serve as the source for files used during the analysis. Click "OK" to finalize the selection.

   Note: DivMelt provides the user with the option of selecting a single .ABT file from a directory for analysis rather than analyzing all files in the directory. This option can be exercised by clicking the "Input file (opt)" button and selecting a specific .ABT file from the navigation window.

   Note: DivMelt also provides the user with the option of restricting the analysis to a subset of the data. This can be a complete single sample name or a portion of a file name. The program will only analyze data in cases where the sample name contains the user-provided character string. If it is desirable to restrict the analysis, enter the desired character string into the blank labeled "Sample name (opt)."

2. Select an output directory by clicking the "Output Options" button then the "Output directory" button and selecting a folder from the navigation window. This folder will receive the files generated during analysis. Click "OK" to finalize the selection.

   Note: DivMelt provides the user with the option of providing an alternative name for the output text file generated during the analysis run. The file name can be entered into the "Output file name (opt)" blank. The user may similarly specify a "Stats output file name (opt)" by specifying the name in the noted blank in the "Output Options" window.

3. Click the "Plotting Options" button, and select the preferred plotting options. DivMelt can generate PDF plots, PNG plots, or no plots based on this selection. Details of these plots are included in the "DivMelt Options, Features, and Outputs" section. Click "OK" to finalize the selection.

4. Click the "Analysis Options" button, and select the preferred analysis options. Default options are as follows: Exclude early peaks, on; Early peak cutoff (degrees C), 3; Exclude late peaks, on; Late peak cutoff (degrees C), 3; Include shoulders, on; Shoulder height threshold (percent), 10; Theta 1 (degrees), 50; Theta 2 (degrees), 30; T1 hold interval (degrees C), 1; T1 slope window (degrees C), 1; Theta 2 (degrees), 30; T2 hold interval (degrees C), 1; and T2 slope window (degrees C), 1. Descriptions of each analysis option

are included in “Section V: DivMelt Options, Features, and Outputs.” Click “OK” to finalize the selection.

Note: Analysis options settings can be invoked from a saved settings file. Details on the save and load settings functions can be found in the section titled “DivMelt Options, Features, and Outputs.”

5. Click “Run analysis” to begin analysis of data following analysis protocol selection. A window will open asking for confirmation and summarizing the settings for the analysis. Click “OK” to begin the analysis. The analysis may take some time to complete depending upon the RAM and processor speed of your machine. A status bar “Progress of analysis…” will appear to indicate the progress of the analysis. Do not modify the R GUI or DivMelt GUI during the analysis. The analysis can be slowed by manipulations of either GUI. Another window will appear when the analysis is complete.

6. When the analysis is complete, view the output directory that was selected for analysis file storage. This directory will contain a set of files depending upon the selections made. By default, a summary text file will be generated. This file will contain many details of the analysis as well as the analysis results themselves. See the following section for a description of the output text files. These include the default summary file noted above plus a statistics file and a summary of rejected samples. Graphics files in the .PDF or .PNG format can also be generated.

SECTION V: DivMelt Options Features and Outputs

The DivMelt graphic user interface (GUI) allows the user to select from a menu of options designed to help identify the temperature at which melting begins and ends (T1 and T2 respectively). These options are then executed following analysis initiation. This section details the various features of DivMelt item by item in the order that they appear on the GUI for DivMelt (Figure 6).

Input Options

The "Input Options" window (Figure 6B) contains options for including data in the analysis and options for controlling how the data outputs are stored.

Input directory: The input directory selection tool is invoked by clicking the button titled "Input directory." This button opens a file selection window that the user may use to select the directory that contains the files to be analyzed.

Input file (opt): The single .ABT file selection tool is invoked by clicking the button titled "Input file (opt)." This button opens a file selection window that the user may use to select the file that will be analyzed.

Sample name (opt): A particular sample name may be selected by typing a sample name or other text into the box adjacent to the tool title. When this tool is used, the analysis is restricted to a subset of the samples. Data are analyzed for all sample names that contain the provided text. This text can be a single sample name or any other text string that is represented in all or a subset of the sample names.

Output Options

The "Output Options" window (Figure 6C) of the graphic user interface contains options for storing the graphical files that are generated as a result of analysis.

Output directory: An output directory can be selected by clicking the button titled "Output directory." This button opens a file selection window that the user may use to select the file that should be used to store all of the files generated during the present analysis.

Output file (opt): A text file is generated for each analysis. This text file is generated in the .csv format and contains the information noted below. By default, this text file will be given the name of the directory that is to be analyzed. The user can supply a specific file name for the text file output. The output file will be stored in the previously selected output directory.

The following information (column labels) is included in the output text file:

1. Plate: name of file from which the data were taken
2. Sample name: name given for sample analyzed
3. Row: row on 96-well plate where sample was loaded
4. Column: column on 96-well plate where sample was loaded
5. T1: temperature when peak plot crosses above Theta 1
6. T2: temperature when peak plot crosses below Theta 2
7. Peak: temperature corresponding to the top of the melting peak
8. Score: HRM Score (T2-T1)
9. theta1: theta value used to assign T1 (increments of 5 degrees)
10. theta2: theta value used to assign T2 (increments of 5 degrees)

11. shouldersOn: This is the status of the shoulder inclusion tool: True/False (was the shoulder inclusion tool in use?)
12. shoulderCutoff: Shoulder inclusion tool cutoff as a proportion of the height of the principal peak (0.1 etc.)
13. earlyOn: Early peak exclusion tool status: True/False (was the early peak exclusion tool in use?)
14. earlyCutoff: Early peak exclusion tool cutoff: Number of degrees that the trace had to remain at baseline between the two peaks before the early peak will be excluded
15. lateOn: Late peak exclusion tool status: True/False (was the late peak exclusion tool in use?)
16. lateCutoff: Late peak exclusion tool cutoff: Number of degrees that the trace had to remain at baseline between the two peaks before the late peak will be excluded
17. t1HldInterval: Number of degrees C over which Theta 1 must be met or exceeded
18. t2HldInterval: Number of degrees C over which Theta 2 must be met or exceeded
19. t1SlpWindow: Number of degrees C over which the slope is measured to determine the T1 candidate temperature
20. t2SlpWindow: Number of degrees C over which the slope is measured to determine the T2 candidate temperature
21. altT1s[1]: Alternative T1 value 1
22. altT1s[2]: Alternative T1 value 2
23. altT1s[3]: Alternative T1 value 3
24. altT2s[1]: Alternative T2 value 1
25. altT2s[2]: Alternative T2 value 2
26. altT2s[3]: Alternative T2 value 3
27. errorCode: cannot find theta/low amplification failure/none (this would include the reason for an error when there was one)
    - 1 – Warning: could not find T1 (T1 reported as zero)
    - 2 – Warning: could not find T2 (T2 reported as zero)
    - 3 – Warning: curve characteristics rejected by Lasso regression
28. Accepted: TRUE/FALSE

Stats output file (opt): The user can specify a name for a summary statistics text file that can be generated to detail the analysis conducted. This statistics file is only generated when a file name is supplied. The stats output file is generated in the .csv format.

The stats output file contains the following information:

1. Sample: sample name
2. min: minimum fluorescence value
3. max: maximum fluorescence value
4. mean: average fluorescence value
5. stddev: standard deviation of fluorescence values
6. sigma: from linear model of fluorescence
7. dmin: minimum delta fluorescence
8. dmax: maximum delta fluorescence
9. dmean: average delta fluorescence
10. dstddev: change in standard deviation

Rejected files text file: All samples that are rejected according to the Lasso model (described in the ‘Analysis Options” section below) are written to a file named “rejected.csv.” There is no option for a user-supplied alternative name for the reject file.

The reject file will contain the following information (columns labeled left to right):

1. Sample: sample name
2. Pred: predicted value used to determine whether data should be accepted or rejected according to the Lasso model file.

Plotting Options

The “Plotting Options” window (Figure 6D) contains options for presenting the graphical files that are generated as a result of analysis.

PDF: The PDF generator is invoked by selecting the bubble to the right of the text “PDF.” When this option is turned on, DivMelt will generate PDF plot outputs that illustrate the shapes of the melting curves and melting peaks. These PDF plots also contain details of the analysis options used and the results of the analysis. Results include T1, T2, Peak, HRM score, and alternative T1/T2 values derived from the data. (See Figure 7 for example PDF plot.)

PNG: The PNG generator is turned on by selecting the bubble to the right of the text “PNG.” When this option is turned on, DivMelt will generate PNG plot outputs that illustrate the shapes of the melting curves and melting peaks. Unlike the PDF plots, the PNG plots contain fewer analysis details. This serves as a cleaner data display that is suitable for presentation and publication.

Show Fluorescence smoothing: This causes the local regression smoothing curve to be included when Fluorescence values are plotted against Temperature (red line Figure 1).

Show dFluorescence smoothing: This causes the local regression smoothing curve to be included when –dFluorescence/dT values are plotted against Temperature. The smoothed curve is used in the analysis regardless of whether this line is displayed (red line Figure 3).

Override X-axis range: This feature allows the user to alter the range of values included on the X-axis of the PDF or PNG plots that are generated.

Analysis Options

The “Analysis Options” window (Figure 6E) contains options for managing various aspects of the analysis.

Exclude early peaks: The exclude early peaks tool is designed to exclude distinct peaks that might occur at significantly lower melting temperatures than the major peak. See Figure 8 for further explanation.

Early peak cutoff (degrees C): This is a component of the exclude early peaks tool. The slider allows the user to select the number of degrees that the trace must return to baseline before the early peak will be excluded.

Exclude late peaks: The exclude late peaks tool is designed to exclude distinct peaks that might occur at significantly higher melting temperatures than the major peak. See Figure 9 for further explanation.

Late peak cutoff (degrees C): This is a component of the exclude late peaks tool. The slider allows the user to select the number of degrees that the trace must remain at baseline after the major peak before the late peak will be excluded.

Include shoulders: The include shoulders tool allows the user to instruct DivMelt to include or exclude shoulders (smaller abutting peaks) that might occur on the low temperature side of principal melting peaks. See Figure 10 for further explanation.

Shoulder height threshold (percent): This is a component of the include shoulders tool. The slider at the right allows the user to select the shoulder height (as a percentage of the height of the principal peak) that is required for the shoulder to be included in the analysis.

Theta 1 (degrees): The slider allows the user to choose the theta value for selection of the T1 value. T1 will be called at the temperature that corresponds to the point where a tangent to the melting peak first rises above a specified angle (Theta 1) with the baseline. See Figure 7 for further explanation.

T1 hold interval (degrees C): The slider allows the user to choose the number of degrees that the Theta 1 angle must be met or exceeded to allow the T1 to be called at a particular point.

T1 slope window (degrees C): The slider can be used to set the number of degrees Celsius over which the slope is measured in an effort to determine the T1 candidate temperature.

Theta 2 (degrees): The slider allows the user to choose the theta value for selection of the T2 value. T2 will be called at the temperature that corresponds to the point where a tangent to the melting peak first falls below a specified angle (Theta 2) with the baseline. See Figure 7 for further explanation.

T2 hold interval (degrees C): The slider allows the user to choose the number of degrees that the Theta 2 angle must be met or exceeded to allow the T2 to be called at a particular point.

T2 slope window (degrees C): The slider can be used to set the number of degrees Celsius over which the slope is measured in an effort to determine the T2 candidate temperature.

HRM score file (opt): A file containing previously calculated HRM score values can be compared with the HRM scores generated during the analysis. Clicking the HRM score file (opt) button opens a file selection window for selection of the HRM score file.

The HRM score file must contain the following information in .csv format (columns labeled from left to right):

1. Plate: name of file from which the data were taken
2. Sample name: name given for sample analyzed
3. Row: row on 96-well plate where sample was loaded
4. Column: column on 96-well plate where sample was loaded
5. T1: temperature when peak plot crosses above Theta 1
6. T2: temperature when peak plot crosses below Theta 2
7. Peak: temperature corresponding to the top of the melting peak
8. Score: HRM Score (T2-T1)

Note: HRM Score comparison files can be easily made by simply deleting all but the first 8 columns of the text summary files generated by DivMelt during a normal analysis run.

Use Lasso regression: The check box at the right is used to invoke the Lasso regression tool. This tool identifies potential analysis failures.

Lasso model file: An alternative Lasso model file may be supplied by the user for use in the analysis. The user-supplied file will be utilized instead of the default file that is included within the application.

The file used to make a Lasso model file must contain the following information in the .csv format (columns labeled from left to right):

1. Sample: sample name
2. Row: row on 96-well plate where sample is located
3. Column: column on 96-well plate where sample is located
4. min: minimum fluorescence value
5. max: maximum fluorescence value
6. mean: average fluorescence value
7. stddev: standard deviation of fluorescence values
8. sigma: from linear model of fluorescence
9. dmin: minimum delta fluorescence
10. dmax: maximum delta fluorescence
11. dmean: average delta fluorescence
12. dstddev: change in standard deviation
13. positive: 1 where positive (example of successful amplification) and 0 when negative (example of amplification failure)

Lasso model file generation
Lasso model files can be generated as described below:

1. Run DivMelt on a set of DNA melting data, and generate a "Stats output" file.

2. Compile a working "Stats output" file. A working "stats output" file can be constructed from a series of DivMelt runs by transferring complete rows from individual "stats output" files to the working stats file. The working stats file should include examples of samples that were not successfully analyzed (PCR failure, other amplification or melting issues) as well as examples of samples that were successfully analyzed.

3. Add a column to the right side of the "Stats output" file. This column should be named "positive."

4. Add a 1 or a 0 to the "positive" column for each row of data included. If the sample on a given row is an example of a positive sample, a "1" should be entered. If the sample on a given row is an example of a negative sample, a "0" should be entered.

5. The working "stats output" file should be saved to the desktop.

6. Open the R GUI or R console, and enter the text below:

   Library(DivMelt)
   modelDivMelt("C:\\Users\\username\\Desktop\\workingfilename.csv","C:\\Users\\username\\Desktop\\newmodel.rda")

Note: The slashes must be doubled "\\" as shown above for R to properly read the command.

Note: This will generate a Lasso model file and save the file to the desktop. The exact locations for both the input (working stats file) and output (new model file) should be specified.

7. To use the newly generated Lasso model file, specify the file within the "Analysis Options" section of the DivMelt GUI.

Note: Enter "?modelDivMelt" in the R GUI after loading the library in R to open documentation describing the routine for creating Lasso model files for DivMelt.

Load Settings

The user may load settings from a text file. This is accomplished by first clicking the "Load Settings" button. This causes an open file window to open. After selecting the proper file, click "OK," to invoke the analysis settings contained in the file. When a settings file is loaded, the Plotting Options and Analysis Options are automatically set. The user will still need to specify input and output options.

Save Settings

The user may save a settings file that can be used to store the settings used in a round of analysis. This file is generated without an extension but can be viewed and edited using notepad (WIN) or textedit (MAC). To save settings, click the "Save Settings" button, and supply a file storage location. Next, click "OK" to save the settings file. The settings file contains details of analysis settings specified in the "Plotting Options" and "Analysis Options" windows.

"Save Settings" file contents:

1. sample: sample name
2. plottype: type of plot generated (may specify "PDF" or "PNG")
3. pdfViewer: specifies program used to view the generated PDF files (function works in UNIX only – acroread=acrobat)
4. pngViewer: specifies program used to view the generated PNG files (function works in UNIX only)
5. smoothFluor: a value of "1" instructs the DivMelt to draw a smoothing line for the Fluorescence vs. Temperature graph, and a value of "0" turns this function off.
6. smoothdFluor: a value of "1" instructs the DivMelt to draw a smoothing line for the dFluorescence vs. Temperature graph, and a value of "0" turns this function off.
7. excludeEarly: excludes early peaks that occur at far lower melting temperatures than the principal peak ("1" tool is on; "0" tool is off)
8. earlyCutoff: value specified in degrees C for deviation between smaller peak and principal peak required for the early peak to be ignored
9. excludeLate: excludes late peaks that occur at higher melting temperatures than the principal peak ("1" tool is on; "0" tool is off)
10. lateCutoff: value specified in degrees C for deviation between smaller peak and principal peak required for the late peak to be ignored

11. includeShoulders: includes shoulders that occur at lower melting temperatures than the principal peak (“1” tool is on; “0” tool is off)
12. shoulderHeight: specifies the shoulder height as a percentage of the height of the principal peak that must be exceeded for the shoulder to be included in the calculation of the HRM score
13. useLasso: the Lasso model tools is turned on by entering “1” and turned off by entering “0”.
14. lassoModel: specifies the Lasso model file name and location when a user-provided file is used in an analysis
15. theta1: sets the theta 1 angle for use in selecting T1 values
16. theta1Hold: specifies the hold interval in degrees C for data used in calculating the T1
17. theta1Window: specifies the window width in degrees C for data used in calculating the T1
18. theta2: sets the theta 2 angle for use in selecting T2 values
19. theta2Hold: specifies the hold interval in degrees C for data used in calculating the T2
20. theta2Window: specifies the window width in degrees C for data used in calculating the T2
21. useXrange: this function turns on or off the user-specified X-axis range function. A “0” corresponds to off, and a “1” corresponds to on.
22. minx: specifies the minimum X value when an alternative X-axis range is specified
23. maxX: specifies the maximum X value when an alternative X-axis range is specified

Show Settings

The user may display all settings that have been specified. If the “Show Settings” button is clicked, a window will appear, displaying the selected options (Figure 6F). Settings are also displayed upon clicking “Run Analysis” (similar to Figure 6F).

SECTION VI: References

1. Cousins MM, Laeyendecker O, Beauchamp G, Brookmeyer R, Towler WI, et al. (2011) Use of a high resolution melting (HRM) assay to compare gag, pol, and env diversity in adults with different stages of HIV infection. PLoS ONE 6: e27211.
2. Cousins MM, Ou SS, Wawer MJ, Munshaw S, Swan D, et al. (2012) Comparison of a high-resolution melting assay to next-generation sequencing for analysis of HIV diversity. J Clin Microbiol 50: 3054-3059.

SECTION VII: Figures and Captions

Figure 1. Melting curve generation.

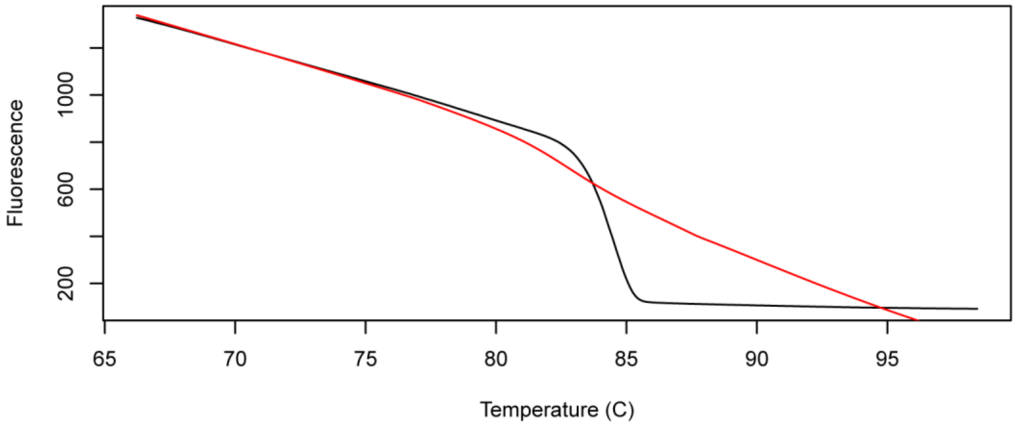

Melting curves (black line) are generated by graphing Fluorescence against Temperature. Fluorescence declines as the DNA melts. DNA melting is visualized through the use of a saturating duplex-dependent DNA intercalating dye (LCGreen Plus). As the DNA melts, the dye is released; unbound dye does not fluoresce (Figure 2). The red line is a local smoothing line that provides additional insight into the melting pattern of the DNA. This line is not used in further calculations.

Figure 2. Melting peak.

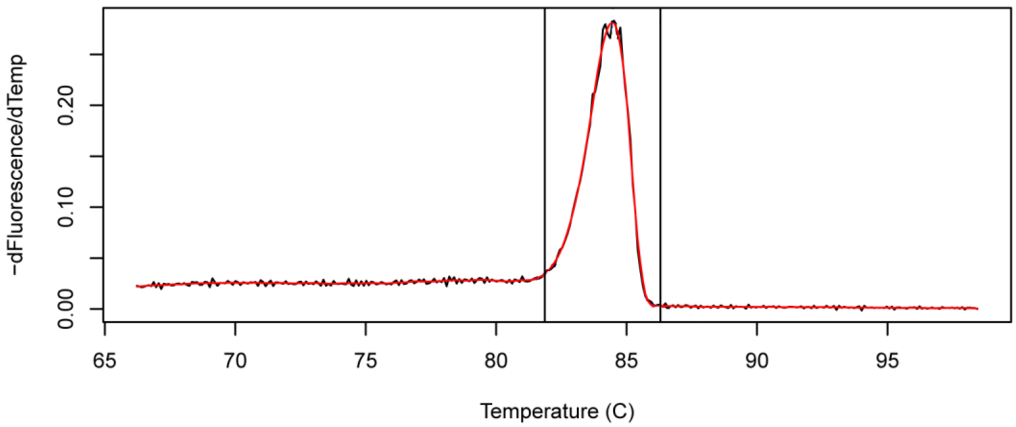

Melting peaks are generated by taking the negative derivative of Fluorescence with respect to Temperature (-dF/dT) and graphing these values against Temperature (black line). The melting peak is derived directly from the melting curve (Fluorescence vs. Temperature; Figure 1). A local smoothing curve is then drawn (red line). The HRM score is calculated by determining the width of the melting peak.

Figure 3. DNA melting and fluorescence.

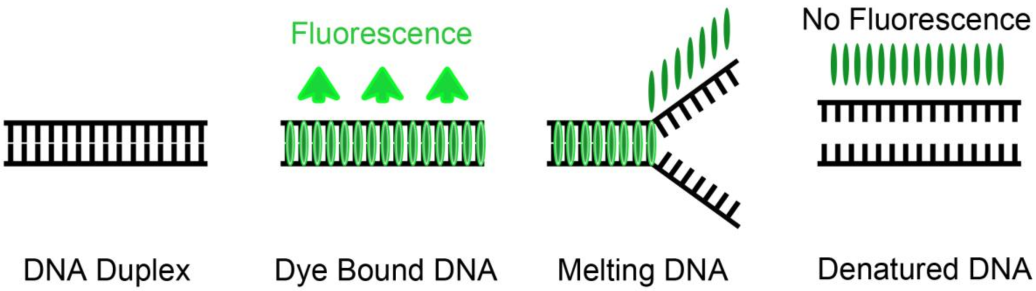

PCR is conducted in the presence of a saturating duplex-dependent fluorescent dye. This dye binds heteroduplex DNA upon completion of the PCR reaction. The dye-bound DNA is then melted on the LightScanner instrument. As the dye is released, fluorescence declines. This allows the melting pattern of the DNA to be tracked with high-resolution (Figure 1).

Figure 4. Goniometer.

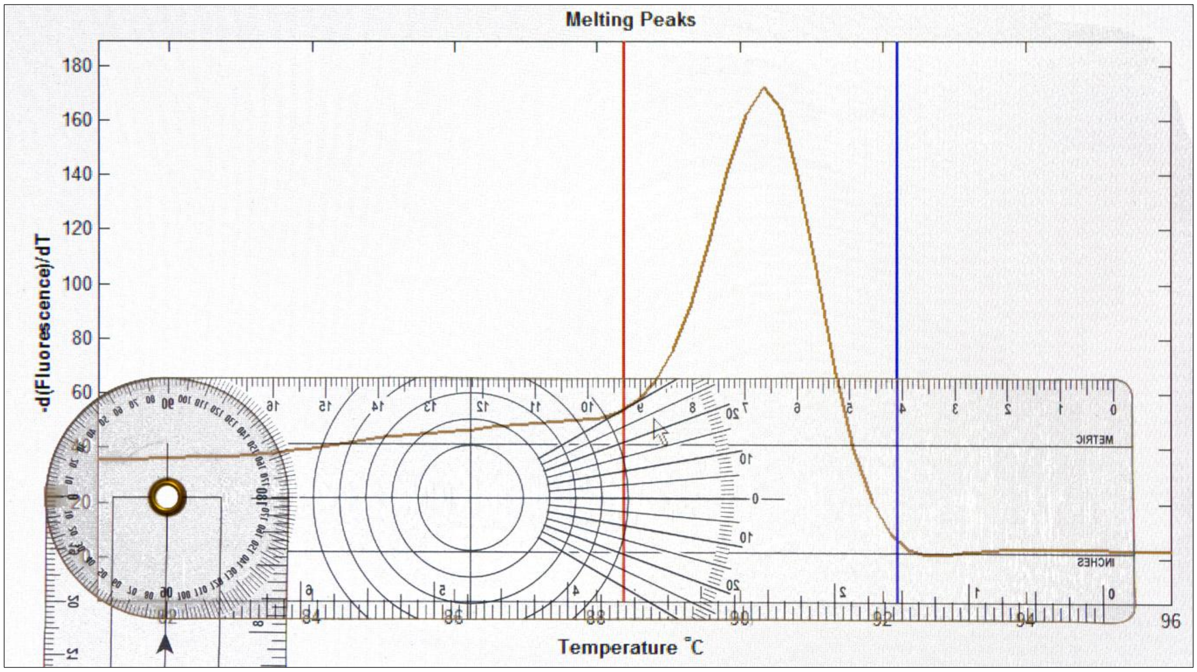

In the past, HRM diversity assay data have been manually analyzed with the aid of a goniometer. The goniometer was pressed to the screen, and the locations at which the tangent to the melting peak curve first rose above and fell below a 30 degree angle with the baseline were determined to be T1 and T2, respectively. In this image, the goniometer is being used to measure a 30 degree angle to identify T1. The red vertical line marks the location that was called using the goniometer (positioned with mouse). The process would then be repeated for T2 to identify a second 30 degree angle and move the blue line to the corresponding location with the mouse.

<u>Figure 5. Preparing data for analysis.</u>

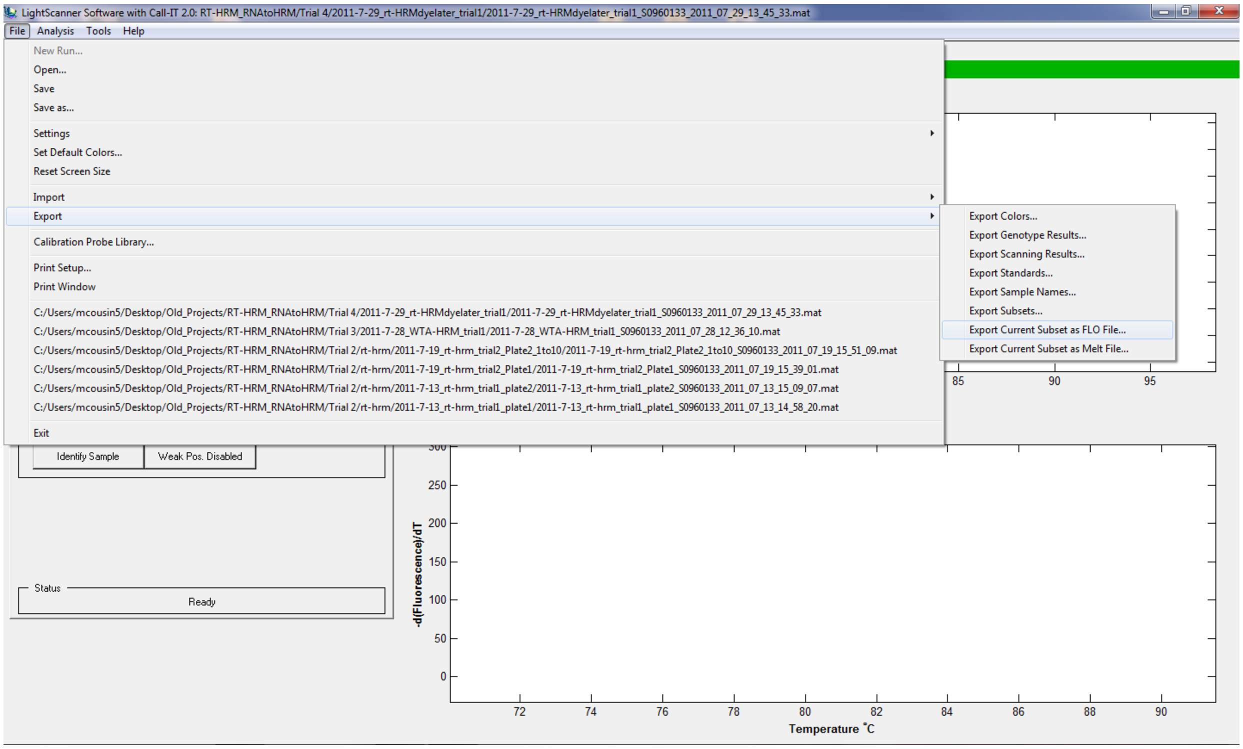

High resolution melting (HRM) curves are generated using the LightScanner instrument. Within the LightScanner Instrument and Analysis Software (BioFire Diagnostics, Salt Lake City, UT), data are labeled with sample names. Data are then exported. This screenshot demonstrates the process of exporting analysis files from the LightScanner software (File→Export→Export Current Subset as FLO File). The export process generates three files (.ABT, .FLO, and .TEM) in the target directory.

Figure 6. DivMelt Graphic User Interface (GUI).

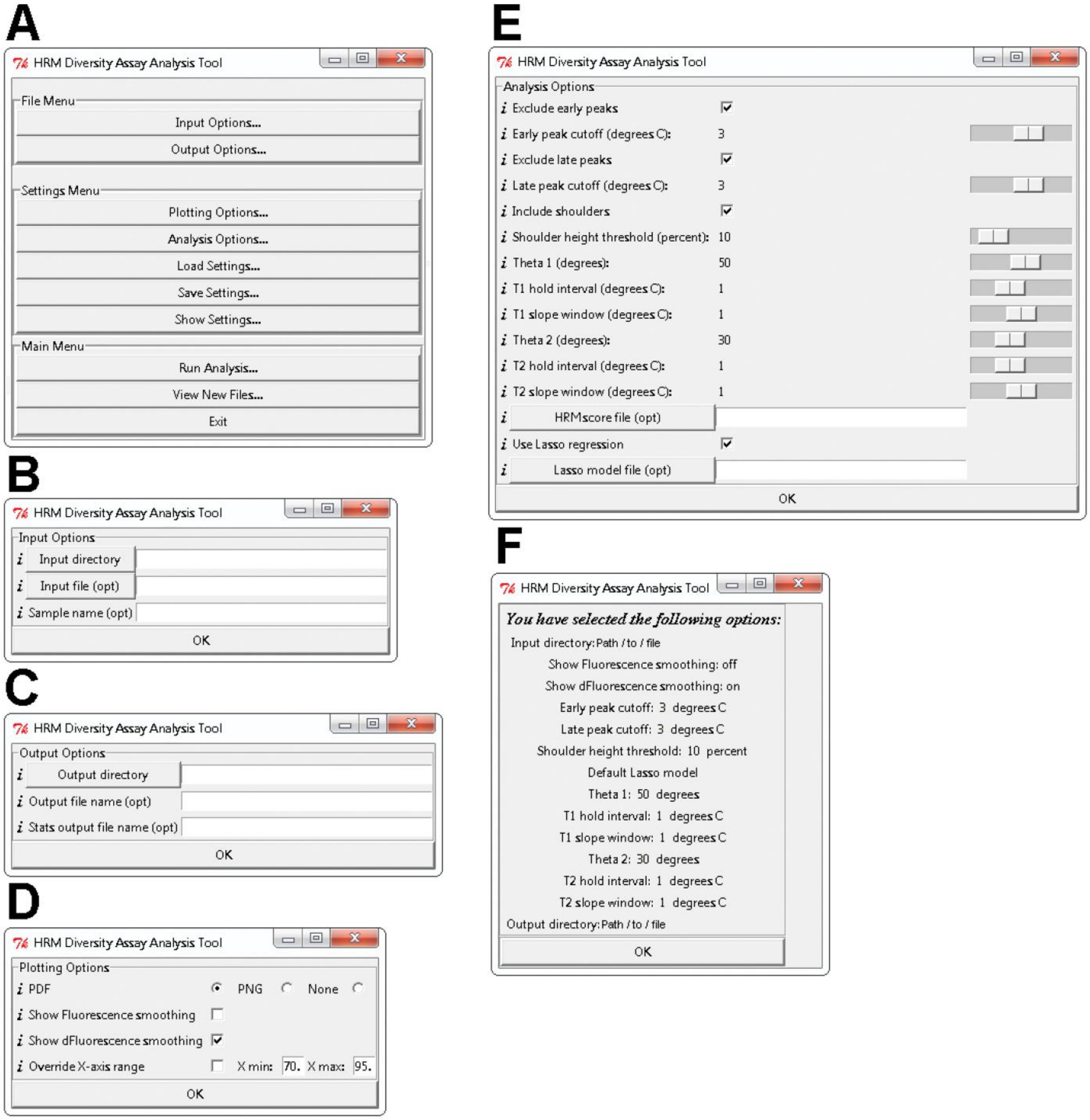

Upon opening DivMelt, various options are available to the user. (A) The initial GUI window displays the “File Menu”, “Settings Menu” and “Main Menu.” Sub-windows relating to file management may be opened from the “File Menu” window. These include (B) the “Input Options” window and (C) the “Output Options” window. From the “Settings Menu” window, additional options can be selected. These include (D) the “Plotting Options” window, (E) the “Analysis Options” window, and (F) the “Show Settings” window. The “Main Menu” is used to initiate analyses. Clicking “Run Analysis” opens the “Review Settings” window (similar to F). See the “DivMelt Options, Features, and Outputs” section for a description of the various tools accessible from the GUI. At the left of each row for items B-E, the “*i*” may be clicked to open a comment box that describes the function of the tool in question. These windows are shown as they display in Windows 7. Note that the windows will display slightly differently in other operating systems.

Figure 7. Fluorescence vs. Temperature and -dFluorescence/dTemperature vs. Temperature plots.

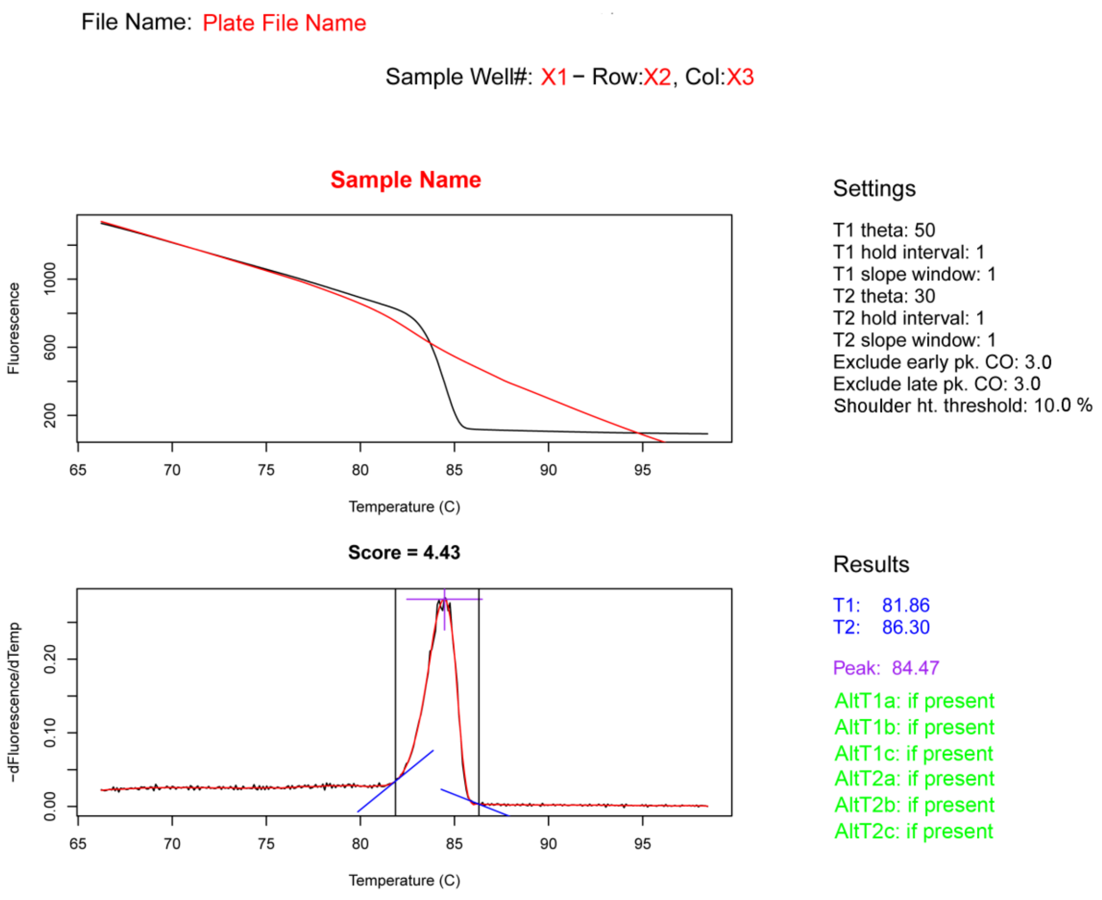

PDF plots are generated as a method to visualize the melting data. There are many features of these plots. The plate file name is noted at the top of each page. Below the file name is the sample location information. These include the sample well number (X1; ranging 1-96), the row letter (X2; ranging A-H), and the column designation (X3; ranging 1-12). The sample name is presented in bold above the Fluorescence vs. Temperature plot (top panel). The Fluorescence vs. Temperature plot tracks the decline in fluorescence as the DNA melts in response to the temperature increase (Figure 1). To the right of this panel, the settings used in the analysis are noted. See the “DivMelt Options, Features, and Outputs” section for descriptions of each function. The lower panel consists of the melting peak or –dFluorescence/dT vs. Temperature plot (Figure 3). To the right of the lower panel are the analysis results (marked on melting peak in equivalent colors). These include the chosen T1 and T2 values (in blue) and the temperature that corresponds to the peak (in purple). Up to three alternative T1 and T2 values may be presented if they are present in the analysis (in green). These values are additional values that met the theta value criteria for T1 and T2 calling but were not selected by DivMelt. The HRM score (Score), the diversity output of the assay, is noted between the top and bottom graphic panels of the figure. This value is calculated by subtracting T1 from T2. This value corresponds to the peak width.

Figure 8. Exclude early peaks tool.

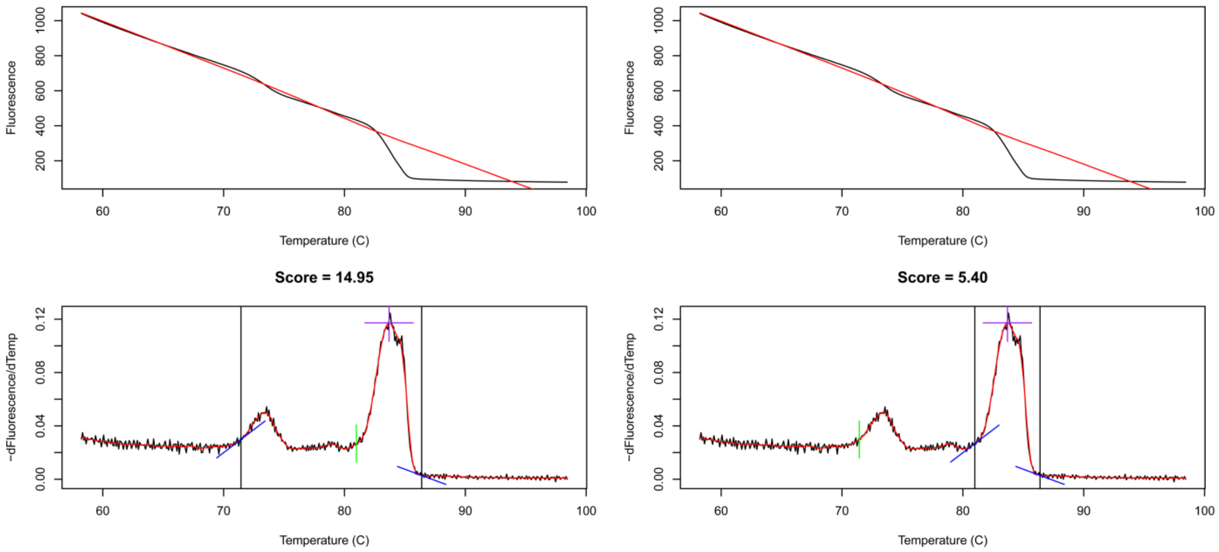

This figure provides a demonstration of the early peak exclusion tool. The tool was developed to avoid including the initial peak that was observed in this sample. In the plot at the left, the tool is "off", and the initial peak that occurs at a lower temperature than the principal peak is included in the HRM score calculation, resulting in a high HRM score. In the plot at the right, the tool is "on," and the low temperature peak is not included in the HRM score calculation. This tool also allows the user to specify which secondary peaks to include based upon the number of degrees C separating the secondary peak from the principal peak.

Figure 9. Exclude late peaks tool.

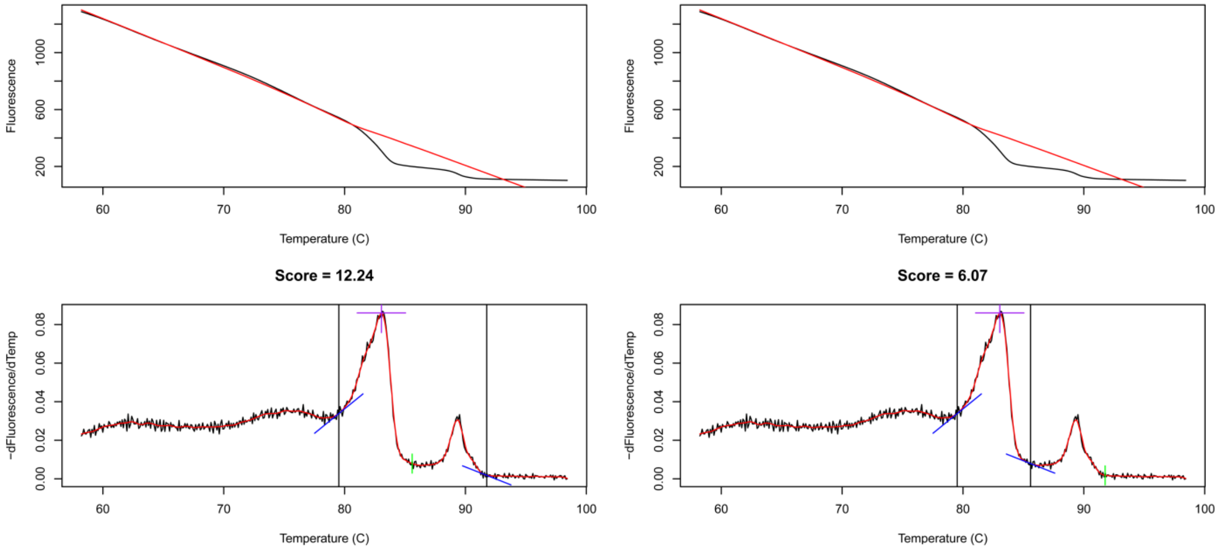

A high temperature peak occurs in this sample. The late peak exclusion tool was developed to allow the user to exclude high temperature secondary peaks from the analysis. In the plot at the left, the tool is “off,” and the late peak is included in the analysis. In the plot on the right, the tool is “on,” and the late peak is excluded from the analysis. This tool also allows the user to specify which secondary peaks to include/exclude based on the distance (degrees C) between the principal peak and the secondary peak.

Figure 10. Include shoulders tool.

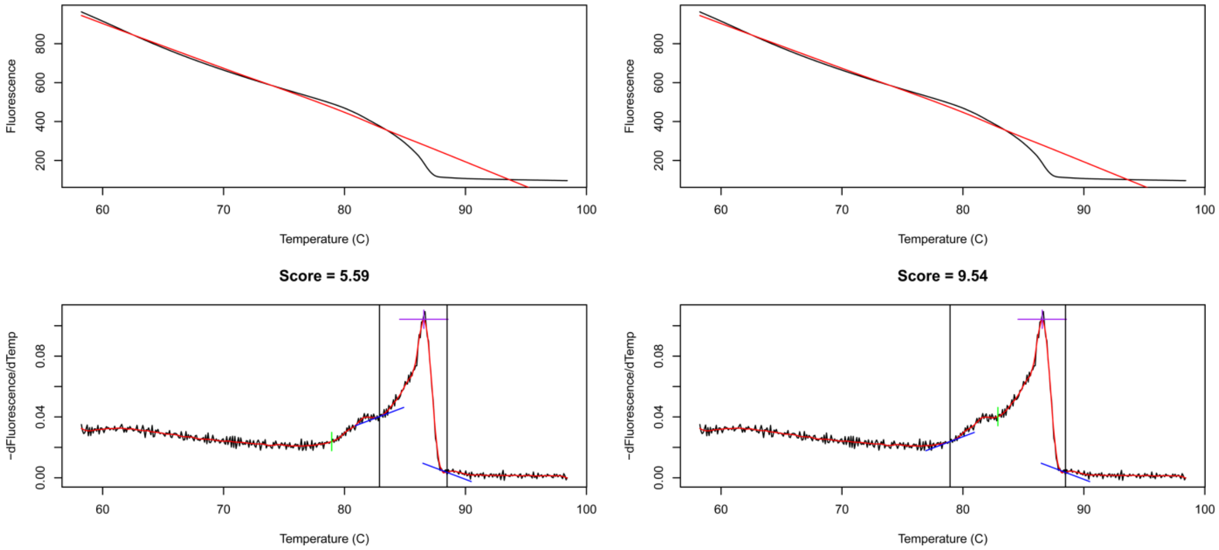

Many peaks that are generated using the HRM diversity assay have shoulders (smaller peaks that are connected to the principal peak) on the low temperature side of the principal peak. In some applications it may be prudent to include the shoulder in calculations, while in others it may be more suitable to exclude them. At left, the shoulder inclusion tool is turned “off.” Note that in the left plot, the shoulder tail is not included in the calculation. At right, the tool is turned “on,” and the shoulder tail is included in the calculation. This tool also allows the user to specify which shoulders to include or exclude based on the height of the shoulder relative to the height of the principal peak.
