# Supplementary material for: Analysis of HIV Using a High Resolution Melting (HRM) Diversity Assay: Automation of HRM Data Analysis Enhances the Utility of the Assay for Analysis of HIV Incidence
Source: PLoS One. 2012 Dec 11;7(12):e51359. doi: 10.1371/journal.pone.0051359 (PMC3519891; doi:10.1371/journal.pone.0051359)
Supplement: Document S2 — Identification of the best HRM Diversity Assay Analysis Tool (DivMelt) protocols for selection of T1 and T2. A large number of different software analysis protocols were evaluated for selection of T1 and T2 (48 protocols for determination of T1 and 16 protocols for determination of T2). The best T1 and T2 selection protocols were those that maximized discrimination between recent and non-recent infection while also minimizing data exclusion due to quality control measures. (PDF) [file pone.0051359.s003.pdf]

Document S2. Identification of the best HRM Diversity Assay Analysis Tool (DivMelt) protocols for selection of T1 and T2.

The purpose of these analyses was to evaluate the performance of different software settings used to identify T1 and T2 in HRM melting curve data. Each combination of four settings was defined as a protocol. Comparisons included 48 protocols for determination of T1 and 16 protocols for determination of T2.

Two criteria were used to select the best protocol for each region in the HIV genome. First, protocols were ranked based on their ability to discriminate between recent and non-recent infection using normalized distance (ND) analysis (see text). Second, the best protocol among the top 10 T1 protocols or 6 T2 protocols based on ND was selected based on the number of test samples that were excluded because duplicate HRM scores did not meet quality control standards (see text). For each genomic region, a “minimum” was established that represented the smallest number of samples excluded by any of the protocols tested. Thresholds were established for identifying the best T1 and T2 selection protocols. For T1 selection protocols, the best protocol must exclude fewer than “minimum + 3” samples; for T2 protocols, the best protocol must exclude the “minimum” number of samples.

The tables included in this document contain the top 10 T1 selection protocols and the top 6 T2 selection protocols as determined by ND analysis; the best protocol for selection of T1 or T2 for each genomic region is also noted (shaded). The dataset used for the ND analysis included 102 samples from individuals with recent HIV infection and 67 samples from individuals with non-recent infection. Data from 20 acute infection samples was added to the dataset for the analysis designed to minimize exclusion of data because duplicate HRM scores did not meet quality control standards; the additional data strengthened the quality of conclusions drawn from this analysis.

<u>T1 selection protocols (Table S1-S6).</u> See text above. For these analyses, T2 settings were held constant (Theta 2 = 30, HI = 0, and SW = 1).

<u>T2 selection protocols (Table S7-S12).</u> See text above. For these analyses, T1 settings were held constant (Theta 1 = 50, HI = 0, and SW = 1).

<u>Summary of derivation of optimal analysis protocols:</u> For each genomic region in HIV, the best T1 selection protocol was combined with the best T2 selection protocol to yield optimal analysis protocols. These T1/T2 combination optimal analysis protocols are presented in Table 1 of the main article.

<u>Footnotes for tables:</u>
The following abbreviations were used in the tables in this supplemental file: ND – normalized distance; HI – hold interval; SW – slope window; SH – shoulder ht. threshold tool (“off” signifies that the tool is not in use).

Table S1. Identification of best T1 selection protocol for GAG1.

ND rank	Theta 1	HI	SW	SH	ND Values	Number excluded [a]
1	30	1	1	10%	1.40573	1
1	30	1	1	0%	1.40573	1
3	60	0	0.5	10%	1.40202	2
4	40	0	0.5	10%	1.38400	3
5	60	0	0.5	0%	1.38058	4
6	40	0	0.5	0%	1.37796	9
7	30	0	1	10%	1.36210	3
8	30	0	1	0%	1.35373	4
9	50	0	0.5	10%	1.35163	1
10	40	1	1	10%	1.34877	1
10	40	1	1	0%	1.34877	1

[a] The number of samples that were excluded by the analysis protocol; the smallest number of samples excluded was zero (“minimum” = 0).

Table S2. Identification of best T1 selection protocol for GAG2.

ND rank	Theta 1	HI	SW	SH	ND Values	Number excluded [a]
1	40	0	1	0%	1.61255	17
2	50	0	1	10%	1.55903	9
2	50	0	1	0%	1.55903	9
4	40	0	1	10%	1.55682	16
5	30	1	0.5	10%	1.53624	17
5	30	1	0.5	0%	1.53624	17
7	40	1	1	10%	1.50239	3
7	40	1	1	0%	1.50239	3
9	40	1	0.5	10%	1.47792	11
9	40	1	0.5	0%	1.47792	11

[a] The number of samples that were excluded by the analysis protocol; the smallest number of samples excluded was two (“minimum” = 2).

Table S3. Identification of best T1 selection protocol for POL.

ND rank	Theta 1	HI	SW	SH	ND Values	Number excluded [a]
1	60	1	0.5	10%	1.80060	1
1	60	1	0.5	0%	1.80060	1
3	50	1	1	10%	1.78094	0
3	50	1	1	0%	1.78094	0
5	60	1	1	10%	1.77737	1
5	60	1	1	0%	1.77737	1
7	50	1	0.5	10%	1.66274	0
7	50	1	0.5	0%	1.66274	0
9	60	0	0.5	0%	1.63362	0
9	60	0	0.5	10%	1.63362	0

[a] The number of samples that were excluded by the analysis protocol; the smallest number of samples excluded was zero (“minimum” = 0).

Table S4. Identification of best T1 selection protocol for ENV1.

ND rank	Theta 1	HI	SW	SH	ND Values	Number excluded [a]
1	60	1	0.5	off	1.61230	1
2	50	1	0.5	off	1.53982	0
3	50	1	1	off	1.52673	0
4	50	0	1	off	1.52443	0
5	60	1	0.5	10%	1.50258	0
5	60	1	0.5	0%	1.50258	0
7	60	0	0.5	off	1.50192	0
8	60	0	1	10%	1.50061	0
8	60	0	1	0%	1.50061	0
10	60	0	0.5	10%	1.49457	0

[a] The number of samples that were excluded by the analysis protocol; the smallest number of samples excluded was zero (“minimum” = 0).

Table S5. Identification of best T1 selection protocol for ENV2.

ND rank	Theta 1	HI	SW	SH	ND Values	Number excluded [a]
1	60	1	0.5	10%	1.06341	7
1	60	1	0.5	0%	1.06341	7
3	60	1	1	10%	1.00521	3
3	60	1	1	0%	1.00521	3
5	60	1	1	off	0.87095	4
6	60	0	1	10%	0.75003	4
6	60	0	1	0%	0.75003	4
8	60	1	0.5	off	0.73390	7
9	60	0	0.5	10%	0.58626	8
10	60	0	0.5	0%	0.57342	9

[a] The number of samples that were excluded by the analysis protocol; the smallest number of samples excluded was one (“minimum” = 1).

Table S6. Identification of best T1 selection protocol for ENV3.

ND rank	Theta 1	HI	SW	SH	ND Values	Number excluded [a]
1	30	0	0.5	0%	1.82582	7
2	30	0	1	0%	1.82515	2
3	30	0	0.5	10%	1.81299	3
4	30	0	1	10%	1.80634	0
5	40	0	0.5	0%	1.78436	5
6	40	0	0.5	10%	1.77271	4
7	30	1	0.5	10%	1.73851	1
7	30	1	0.5	0%	1.73851	1
9	40	0	1	0%	1.73346	1
9	40	0	1	10%	1.73346	1

[a] The number of samples that were excluded by the analysis protocol; the smallest number of samples excluded was zero (“minimum” = 0).

Table S7. Identification of best T2 selection protocol for analysis of GAG1.

ND rank	Theta 2	HI	SW	ND Values	Number excluded [a]
1	60	0	0.5	1.38721	1
2	60	0	1	1.37029	1
2	60	1	1	1.37029	1
4	60	1	0.5	1.36854	1
5	50	0	1	1.36144	1
5	50	1	1	1.36144	1

[a] The number of samples that were excluded by the analysis protocol; the smallest number of samples excluded was one (“minimum” = 1).

Table S8. Identification of best T2 selection protocol for analysis of GAG2.

ND rank	Theta 2	HI	SW	ND Values	Number excluded [a]
1	30	0	0.5	1.64811	12
2	60	1	0.5	1.60512	10
3	50	0	1	1.59728	10
4	40	0	1	1.57331	8
5	60	0	0.5	1.57311	14
6	30	1	0.5	1.56570	9

[a] The number of samples that were excluded by the analysis protocol; the smallest number of samples excluded was eight (“minimum” = 8).

Table S9. Identification of best T2 selection protocol for analysis of POL.

ND rank	Theta 2	HI	SW	ND Values	Number excluded [b]
1	60	0	0.5	1.62078	1
2	50	0	0.5	1.58615	0
2	50	1	0.5	1.58615	0
4	60	1	0.5	1.58378	0
5	50	0	1	1.57736	0
5	50	1	1	1.57736	0

[a] The number of samples that were excluded by the analysis protocol; the smallest number of samples excluded was zero (“minimum” = 0).

Table S10. Identification of best T2 selection protocol for analysis of ENV1.

ND rank	Theta 2	HI	SW	ND Values	Number excluded [a]
1	40	0	0.5	1.44268	0
1	40	1	0.5	1.44268	0
3	30	0	1	1.42833	0
3	30	1	1	1.42833	0
5	30	0	0.5	1.42684	0
5	30	1	0.5	1.42684	0

[a] The number of samples that were excluded by the analysis protocol; the smallest number of samples excluded was zero (“minimum” = 0).

Table S11. Identification of best T2 selection protocol for analysis of ENV2.

ND rank	Theta 2	HI	SW	ND Values	Number excluded [a]
1	30	0	0.5	0.42268	8
1	30	1	0.5	0.42268	8
3	40	0	0.5	0.41054	7
3	40	1	0.5	0.41054	7
5	50	0	0.5	0.40235	7
5	50	1	0.5	0.40235	7

[a] The number of samples that were excluded by the analysis protocol; the smallest number samples excluded was seven (“minimum” = 7).

Table S12. Identification of best T2 selection protocol for analysis of ENV3.

ND rank	Theta 2	HI	SW	ND Values	Number excluded [a]
1	60	0	1	1.64231	0
1	60	1	1	1.64231	0
3	50	0	1	1.64190	0
3	50	1	1	1.64190	0
5	40	0	1	1.63662	0
5	40	1	1	1.63662	0

[a] The number of samples that were excluded by the analysis protocol; the smallest number of samples excluded was zero (“minimum” = 0).
