# Supplementary material for: Analysis of HIV Using a High Resolution Melting (HRM) Diversity Assay: Automation of HRM Data Analysis Enhances the Utility of the Assay for Analysis of HIV Incidence
Source: PLoS One. 2012 Dec 11;7(12):e51359. doi: 10.1371/journal.pone.0051359 (PMC3519891; doi:10.1371/journal.pone.0051359)
Supplement: Document S3 — Accuracy of T1 and T2 values identified using the HRM Diversity Assay Analysis Tool (DivMelt). T1 and T2 temperatures identified with DivMelt were visually scored for accuracy. (PDF) [file pone.0051359.s004.pdf]

Document S3. Accuracy of T1 and T2 values identified using the HRM Diversity Assay Analysis Tool (DivMelt).

T1 (start melt) and T2 (end melt) temperatures were identified using DivMelt. These values were compared to T1 and T2 values determined by visual inspection of melt curves. T1 and T2 values that clearly did not correspond to the start and end of melting were defined as improperly identified. These tables show the percentage of melt curves with improperly identified T1 and T2 temperature values (see text). Note: all peak temperature values were properly identified by all analysis protocols.

Table S13. Percentage of T1 values that were improperly identified using the optimal analysis protocol for each region of the HIV genome[a].

**Region Used for Optimization**[b]	**Region Analyzed**[c]					
	GAG1	GAG2	POL	ENV1	ENV2	ENV3
GAG1	0.53	5.29	0.00	0.00	0.79	0.53
GAG2	0.00	0.26	0.00	0.00	0.26	0.53
POL	0.00	1.32	0.53	0.00	0.00	0.00
ENV1	3.17	5.03	3.70	0.79	2.12	3.44
ENV2	0.53	1.59	0.53	0.00	0.00	0.79
ENV3	0.79	23.28	0.79	0.00	0.53	0.79
All regions	0.00	0.00	0.53	0.00	0.00	0.00

[a] All values reported as percentages. The region used to optimize each protocol is shown in the column on the left; the regions analyzed are shown in the headers for the six other columns.
[b] Detailed region-specific analysis protocol descriptions are shown in Table 1.
[c] HIV regions are described in the footnote for Table 1.

Table S14. Percentage of T2 values that were improperly identified using the optimal analysis protocol for each region of the HIV genome[a].

**Region Used for Optimization**[b]	**Region Analyzed**[c]					
	GAG1	GAG2	POL	ENV1	ENV2	ENV3
GAG1	0.53	17.20	0.26	0.26	0.00	0.53
GAG2	0.00	1.32	0.00	0.00	0.00	0.53
POL	0.00	10.58	0.00	0.00	0.00	0.53
ENV1	0.00	7.94	0.53	0.00	0.00	0.53
ENV2	0.00	7.67	0.00	0.00	0.00	0.79
ENV3	0.00	2.38	0.00	0.00	0.00	0.00
All regions	0.00	1.59	0.00	0.00	0.53	0.53

[a] All values reported as percentages. The region used to optimize each protocol is shown in the column on the left; the regions analyzed are shown in the headers for the six other columns.
[b] Detailed region-specific analysis protocol descriptions are shown in Table 1.
[c] HIV regions are described in the footnote for Table 1.
